# Impact of Skin Exposure to Benzo[a]pyrene in Rat Model: Insights into Epidermal Cell Function and Draining Lymph Node Cell Response

**DOI:** 10.3390/ijms25168631

**Published:** 2024-08-08

**Authors:** Anastasija Malešević, Dina Tucović, Jelena Kulaš, Ivana Mirkov, Dušanka Popović, Maja Čakić Milošević, Aleksandra Popov Aleksandrov

**Affiliations:** 1 Immunotoxicology Group, Department of Ecology, Institute for Biological Research “Siniša Stanković”, National Institute of Republic of Serbia, University of Belgrade, 142 Bulevar Despota Stefana, 11000 Belgrade, Serbia; anastasija.malesevic@ibiss.bg.ac.rs (A.M.); dina.mileusnic@ibiss.bg.ac.rs (D.T.); jelenakulas381@gmail.com (J.K.); mirkovi@ibiss.bg.ac.rs (I.M.); dusanka.popovic@ibiss.bg.ac.rs (D.P.); 2 Institute of Zoology, Faculty of Biology, University of Belgrade, 16 Studentski trg, 11000 Belgrade, Serbia; maja@bio.bg.ac.rs

**Keywords:** epicutaenous benzo[a]pyrene application, skin explants, epidermal cell activity, draining lymph nodes cell activity, rats

## Abstract

The skin is a direct target of the air pollutant benzo[a]pyrene (BaP). While its carcinogenic qualities are well-studied, the immunotoxicity of BaP after dermal exposure is less understood. This study examines the immunomodulatory effects of a 10-day epicutaneous BaP application, in environmentally/occupationally relevant doses, by analyzing ex vivo skin immune response (skin explant, epidermal cells and draining lymph node/DLN cell activity), alongside the skin’s reaction to sensitization with experimental hapten dinitrochlorobenzene (DNCB). The results show that BaP application disrupts the structure of the epidermal layer and promotes immune cell infiltration in the dermis. BaP exposure led to oxidative stress in epidermal cells, characterized by decreased reduced glutathione and increased AHR and Cyp1A1 expression. Production and gene expression of proinflammatory cytokines (TNF, IL-1β) by epidermal cells decreased, while IL-10 response increased. Decreased spontaneous production of IFN-γ and IL-17, along with unchanged IL-10, was observed in DLC cells, whereas ConA-stimulated production of these cytokines was elevated. Local immunosuppression caused by BaP application seems to reduce the skin’s response to an additional stimulus, evidenced by decreased effector activity of DLN cells three days after sensitization with DNCB. These findings provide new insight into the immunomodulatory effects and health risks associated with skin exposure to BaP.

## 1. Introduction

Air pollution is a global health concern affecting billions of people. It is estimated that more than 90% of the world population is exposed to unhealthy air, by living in places that do not comply with the WHO Air Quality Guidelines [1]. Polycyclic aromatic hydrocarbons (PAHs) are common indoor and outdoor environmental air pollutants, whose health effects have been a matter of extensive research. Environmental PAHs include a mixture of different compounds, whose composition and concentration vary significantly depending on their source (such as traffic, wildfire) or environmental factors (such as weather, geography) [2,3]. Benzo[a]pyrene (BaP) is considered a representative PAH, due to its notable and well-studied toxicity, correlations with other PAHs and a high prevalence in the environment, arising from both pollution (such as industrial incineration, smoke and automobile exhaust) and natural burning processes [4,5,6]. Studies focusing solely on BaP toxicity have enabled better understanding of its specific toxicity mechanisms without the confounding effects of other PAHs, and have allowed the introduction of BaP equivalent factors for evaluating the relative carcinogenicity and toxicity of other PAHs [7]. BaP is a well-characterized human carcinogen [8]. Its metabolism results in the formation of reactive metabolites that create DNA adducts and disrupt cell cycles, ultimately resulting in uncontrolled cell proliferation and cancer development [6]. Furthermore, BaP is a potent immunotoxic agent. Although it is generally known to induce immunosuppression, a number of studies showed its immunostimulatory effect [4]. Within a cell, BaP forms a complex with an aryl hydrocarbon receptor (AhR), leading to the activation of this receptor. AhR activation triggers the expression of cytochrome P450 isoforms 1A1 (CYP1A1) and 1B1 (CYP1B1) and consequently BaP metabolism [9]. Activation of AhR could lead to the expression of various other genes, including those involved in inflammation and oxidative stress [9].

Inhalation and ingestion are considered primary ways of exposure to BaP, through contaminated air and water/food, respectively. The skin, being the outermost body organ, is an important target directly exposed to BaP and air pollutants in general. However, it is often underestimated as a site of exposure. Despite daily contact of skin with contaminated air in the general population, BaP-induced dermatotoxicity is primarily considered in the context of occupational exposure. While the carcinogenic effect of BaP on the skin has been recognized and extensively studied, less is known about the immunotoxicity of dermal exposure to BaP, despite a previous study suggesting BaP, among other PAHs, has a greater capacity to disrupt skin barrier function and induce inflammation [10]. Additionally, recent studies in mice have suggested that skin exposure to BaP aggravates psoriasis [11], while oral or systemic exposure to BaP enhances atopic dermatitis [12]. These studies point to the potential of BaP to affect skin immune homeostasis, but the underlying mechanisms are largely missing. 

The aim of this study is to investigate the immunomodulatory effects of BaP in skin following 10-day epicutaneous application of BaP at doses relevant to environmental and low-dose occupational exposure, using a rat model. Despite the differences between rat and human skin (thickness, permeability, hair follicle density, etc.) [13] rat skin is often used as a model system in toxicology studies, mainly due to its availability, ease of use and a large amount of pre-existing literature data that allows comparisons to be made [14]. Additionally, the similar thickness between rat back-skin layers and human skin, especially human skin most exposed to air pollution [13], along with studies indicating that hair follicles negligibly affect uptake of BaP [10], support the use of rats as a valuable model for studying the toxicity of topically applied BaP. The results will provide new data on BaP’s effects on skin immune responses, enhancing the understanding of BaP’s potential impact on human health under real-life skin exposure settings. The study focuses on the ex vivo skin immune response (skin explant, epidermal cells and draining lymph node (DLN) cell activity) as well as on investigation of BaP exposure impact on immune priming in the skin (sensitization to experimental contact allergens). The obtained data showed that BaP-induced dermatotoxicity is associated with reductions in epidermal cell immune function and decreased IFN-γ and IL-17 responses in DLN.

## 2. Results

### 2.1. Topical Application of BaP Induces Skin Damage and Inflammation

**Figure 1:**
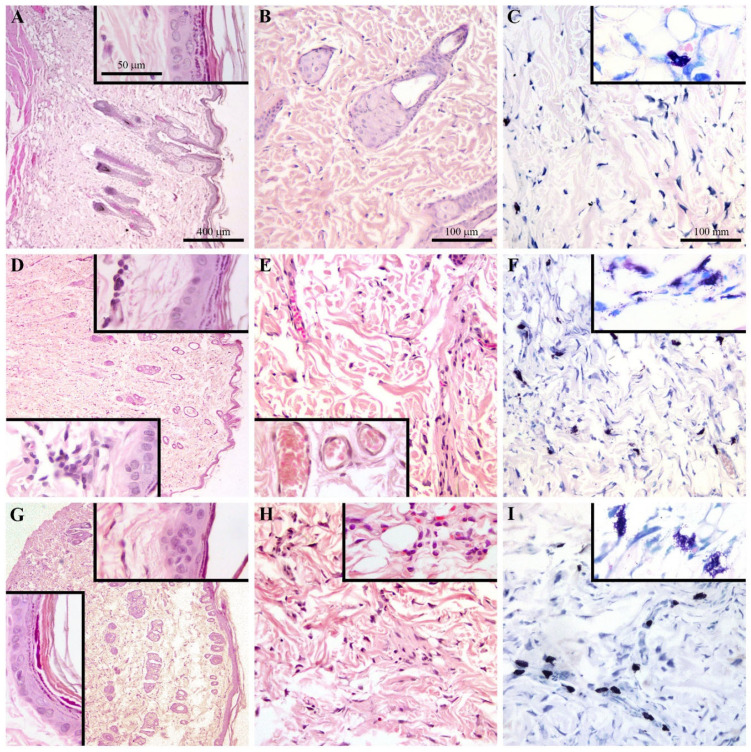
Histology of skin following epicutaneous BaP application. (**A**) Control skin with normal histological structure. Epidermal layer and upper dermis with thin collagen fibrils parallel arranged in control skin (insert). (**B**) Collagen bundles in different directions. (**C**) Mast cells appearance in deep dermis of control skin. (**D**) BaP 20 ng/cm^2^ application induced increased skin thickness, appearance of epidermal cells with pyknotic nuclei (top insert), disturbed epidermal stratification (bottom insert) and dermal infiltration (inserts). (**E**) Increased cellularity, disarrangement of collagen bundles and mild hyperemia of dermal blood vessels (insert), as well as (**F**) more numerous and densely stained mast cells in dermis following BaP 20 ng/cm^2^ application. (**G**) BaP 100 ng/cm^2^ application resulted in patchy desquamation (bottom insert) and pronounced keratinocytes hyperplasia with atypical nuclei and loss of ordered stratification (top insert). (**H**) More pronounced dermal cellularity and (**I**) mast cells number, mostly located in deep dermis and along blood vessels, in process of degranulation (insert) following BaP 100 ng/cm^2^ application.

Histological analysis of the control skin showed a normal histological structure (Figure 1A–C). The topical application of a lower BaP dose (20 ng/cm^2^) led to an increase in the overall thickness of the skin compared to the control (Figure 1D). Single keratinocytes with pyknotic nuclei (highly condensed) appeared in the basal epidermal layer, and cell stratification was slightly disturbed. The dermis as a whole showed increased cellularity and vascularization compared to the untreated animals (Figure 1E). In the deeper dermis, loosening and disarrangement of collagen bundles and mild hyperemia were observed (Figure 1E). Slight perivascular infiltration, small foci of mononuclears and delamination of collagen fibers were seen in the upper dermis (Figure 1E, insert). At this BaP dose, the mast cells appeared larger, more densely stained and more numerous (Figure 1F), compared to controls. At a higher BaP dose (100 ng/cm^2^), the general histological appearance of the skin resembled that of animals treated with a lower dose. In addition, several other epidermal changes were noted such as intensified patchy desquamation (Figure 1G, bottom insert), more pronounced focal keratinocyte hyperplasia with some atypical nuclei and loss of ordered cell stratification (Figure 1G, top insert), and focal vacuolization/ballooning of keratinocytes. The changes in the dermis generally followed the pattern observed in the lower BaP group, with more pronounced cellularity (Figure 1H). The mast cells in the deep dermis were even more abundant than in the lower dose group, and some of them were also present in the upper dermis. Many mast cells appeared to be degranulated or in the process of degranulation (Figure 1I). See Supplementary Data S1 for original images.

**Figure 2:**
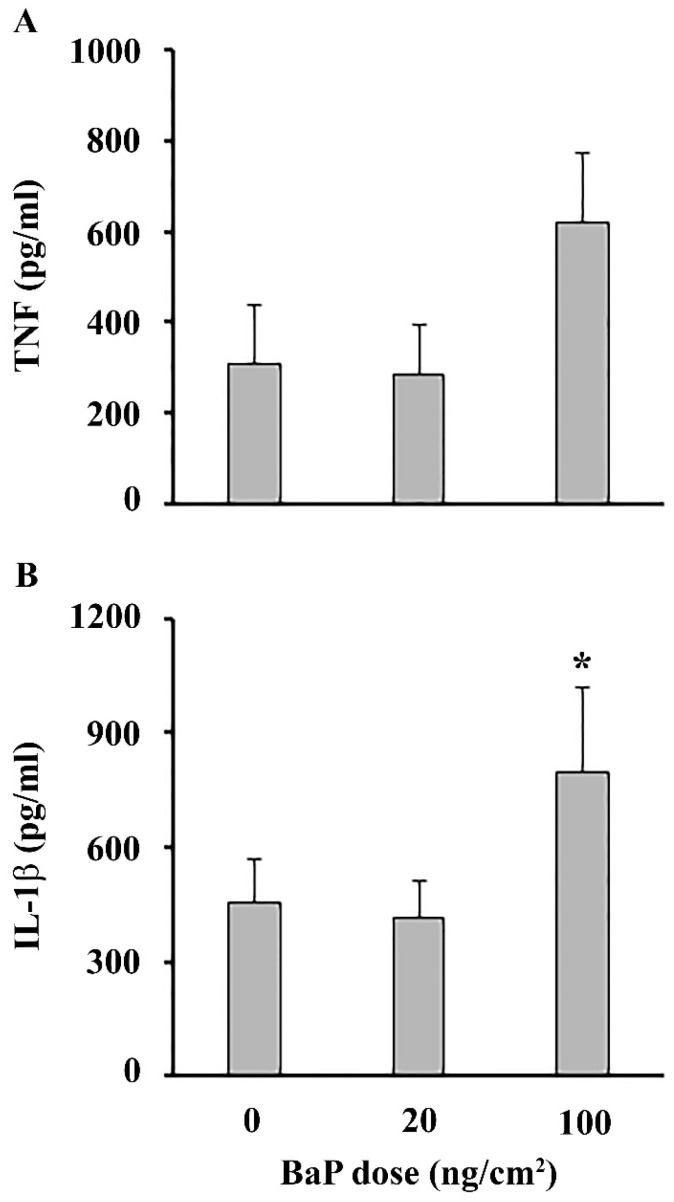
Inflammatory response in skin following epicutaneous BaP application. (**A**) TNF and (**B**) IL-1β production by skin explants. Results are presented as mean (±SD). Significance at: * *p* < 0.05 vs. control animals (BaP dose 0 ng/cm^2^).

Determination of cytokines in the skin-conditioned medium showed increased TNF (*p* = 0.05) (Figure 2A) and IL-1β (Figure 2B), solely at a higher BaP dose. 

### 2.2. Skin Exposure to BaP Triggers Stress and Immunomodulatory Response in Epidermal Cells

**Figure 3:**
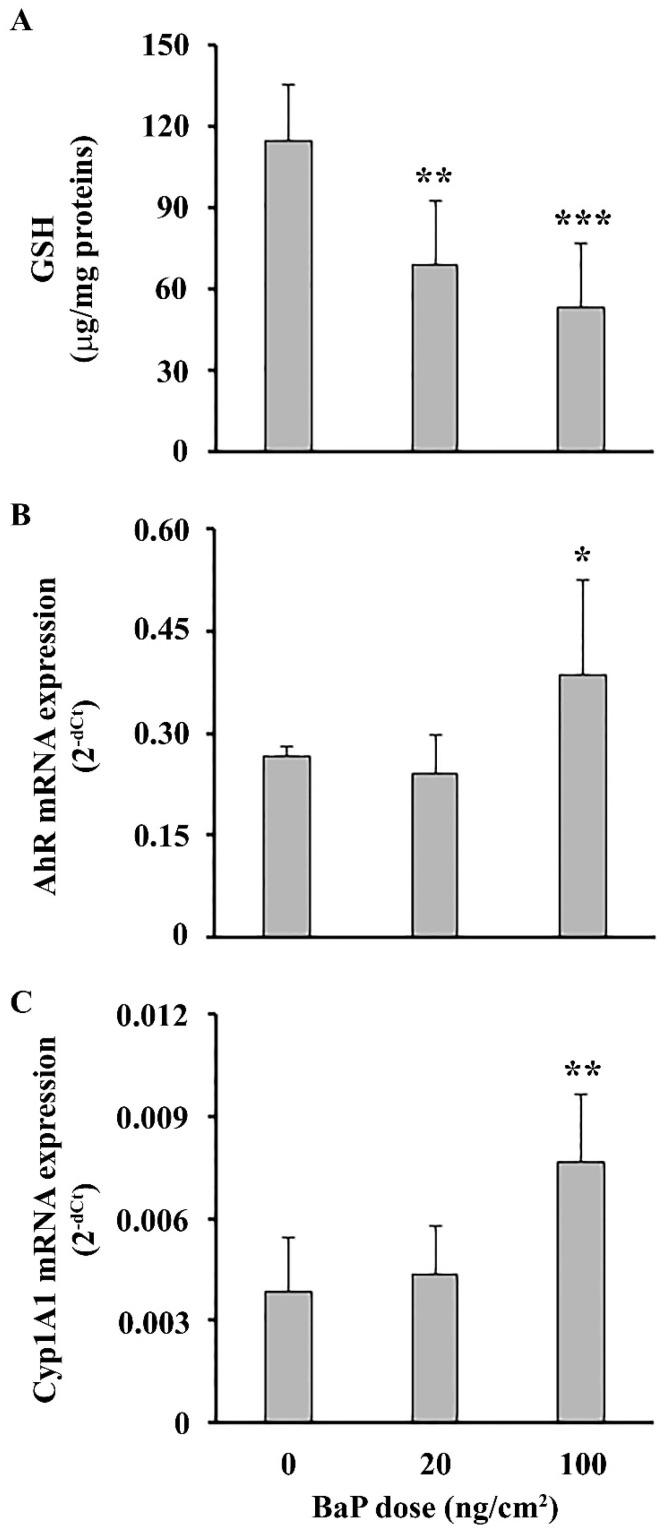
Stress response in epidermal cells following epicutaneous BaP application. (**A**) Intracellular GSH. Epidermal cells mRNA expression of (**B**) AhR and (**C**) Cyp1A1. Results are presented as mean (±SD). Significance at: * *p* < 0.05, ** *p* < 0.01, *** *p* < 0.001 vs. control animals (BaP dose 0 ng/cm^2^).

Topical application of both BaP doses decreased GSH level in epidermal cells (Figure 3A). Analysis of the AhR signaling pathway revealed an elevated mRNA expression for AhR (Figure 3B) and its target gene encoding for drug-metabolizing enzyme Cyp1A1 at the higher dose (Figure 3C). 

**Figure 4:**
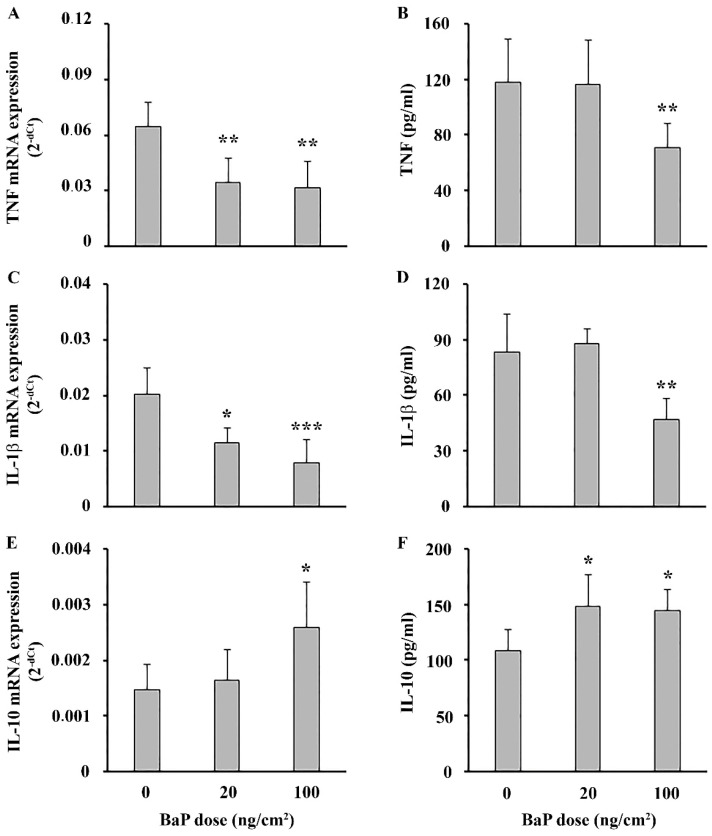
Effect of epicutaneous BaP application on epidermal cells’ cytokine response. (**A**,**B**) TNF, (**C**,**D**) IL-1β and (**E**,**F**) IL-10 mRNA expression and spontaneous production, respectively. Results are presented as mean (±SD). Significance at: * *p* < 0.05, ** *p* < 0.01, *** *p* < 0.001 vs. control animals (BaP dose 0 ng/cm^2^).

The analysis of mRNA expression showed a decrease in TNF and IL-1β at both doses, compared to controls (Figure 4A and Figure 4C, respectively). Similarly, measurements of ex vivo cytokine production by epidermal cells revealed a decrease in the production of TNF and IL-1β at the higher BaP dose (Figure 4B and Figure 4D, respectively). On the other hand, mRNA expression of anti-inflammatory cytokine IL-10 was increased at the higher dose while the production of this cytokine was increased at both doses (Figure 4E,F). No changes in metabolic viability of cultured epidermal cells were detected between the groups (0.153 ± 0.038, 0.148 ± 0.037 and 0.141 ± 0.021 for control, BaP 20 ng/cm^2^ and 100 ng/cm^2^, respectively). 

### 2.3. DLN Cell Response Following Topical BaP Application

As shown in Table 1, topical application of BaP increased the number of DLN cells at a higher dose, compared to controls. The number of viable cells (determined by using a trypan blue exclusion assay) and metabolic viability of freshly isolated cells were unchanged between groups. Phenotypic analysis of DLN T-cell subset composition revealed a decreased relative number of both CD4^+^ cells and CD8^+^ cells at the higher dose. 

**Figure 5:**
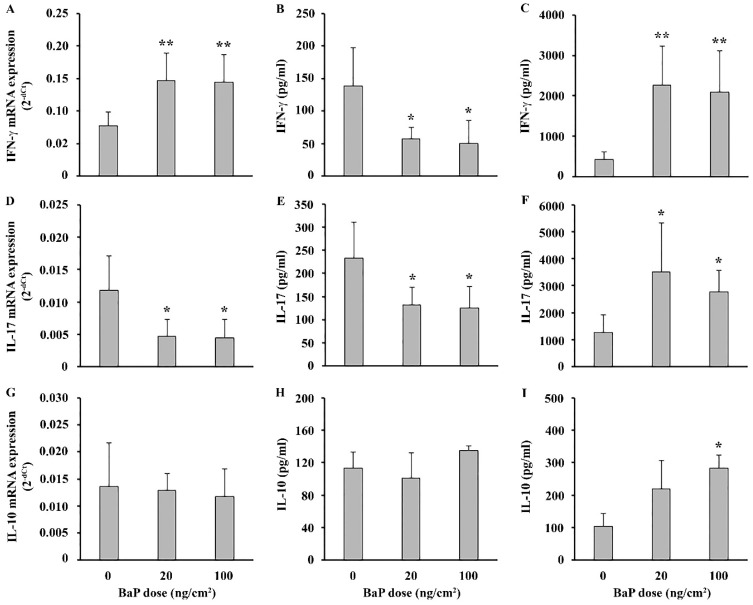
Effect of epicutaneous BaP application on draining lymph node cells’ cytokine response. (**A**–**C**) IFN-γ, (**D**–**F**) IL-17 and (**G**–**I**) IL-10 mRNA expression, spontaneous and ConA-stimulated production, respectively. Results are presented as mean (±SD). Significance at: * *p* < 0.05, ** *p* < 0.01 vs. control animals (BaP dose 0 ng/cm^2^).

Analysis of mRNA expression showed an increase for IFN-γ (Figure 5A) and a decrease for IL-17 at both doses (Figure 5D), while spontaneous production of IFN-γ (Figure 5B) and IL-17 (Figure 5E) by DLN cells was decreased at both doses following BaP application, compared to controls (Figure 5). Following stimulation with ConA, DLN cell production of both cytokines was increased at both doses of BaP (Figure 5C,F). Expression of mRNA and spontaneous production of IL-10 were unchanged, while ConA-stimulated IL-10 production was increased at a higher dose (Figure 5G, Figure 5H and Figure 5I, respectively). 

### 2.4. Effect of Skin Exposure to BaP on Sensitization to DNCB 

In order to assess BaP’s effect on immune priming activity in the skin, skin sensitization with experimental contact allergen DNCB was achieved following topical application of a higher BaP dose, as this dose induced more pronounced immunomodulatory effect in the skin. Our results showed that skin pre-exposure to a higher dose of BaP resulted in a reduced DLN cell number, as well as decreased relative counts of CD4^+^ and CD8^+^ cells in DLN, three days after hapten application (Table 2). BaP application did not compromise cell viability in DLN as it was unchanged between groups, while the metabolic viability of freshly isolated DLN cells was even higher in BaP-treated sensitized animals, compared to sensitized controls. The decrease in CD4^+^ and CD8^+^ cell number was accompanied by decreased spontaneous production of IFN-γ, one of the key cytokines during sensitization. In contrast to IFN-γ, IL-10 production by DLN cells isolated from BaP-treated sensitized animals was increased compared to sensitized control animals (Table 2).

## 3. Discussion

In this study, a local immunomodulatory effect of skin exposure to BaP was examined, by analyzing skin, epidermal cells, DLN cell response and the impact on epicutaneous sensitization with DNCB. Histopathological analysis revealed cellular damage and disturbed structure in the epidermal layer induced by BaP application, characterized by the appearance of pyknotic nuclei in epidermal cells and loss of epidermal stratification. Epidermal damage seems to have been more pronounced following a higher dose application as it was further followed by intensified desquamation and vacuolization in keratinocytes. Hyperplasia of epidermal cells, detected at a higher BaP dose, could indicate proliferation as a regenerative response to the presence of the insult [15]. Decreased thickness and density of collagen fibers further demonstrates the damaging effect of BaP on the skin, causing changes which can decrease skin fragility [16]. This finding may be indicative of BaP’s inhibitory effect on collagen synthesis, as previously shown in human periodontal ligament cells [17]. Additionally, one early study reported that BaP could non-specifically bind to collagen, inhibiting formation of collagen fibrils [18]. Increased infiltration of immune cells in the dermis, together with increased number and activity of mast cells, detected at both BaP doses, might be a response to a tissue injury. BaP-induced infiltration of inflammatory cells has been previously detected in the dermis [10] and other organs such as the lungs and cervix [19,20]. Although data on BaP’s effects on mast cell infiltration and degranulation are limited, a recent study showed that BaP enhances the infiltration and degranulation of mast cells in mite allergen-induced atopic dermatitis (AD)-like skin lesions in mice [21]. Mast cells can coordinate an event such as inflammation as they are abundant producers of growth factors and cytokines. Indeed, increased TNF and IL-1β production by skin explants demonstrates an inflammatory process in the skin, at least following application of a higher BaP dose, which could be associated with the more pronounced infiltration of immune cells and mast cell degranulation at this dose. Moreover, a previous study showed that BaP induces damage in skin fibroblasts and affects pathways associated with skin inflammation [6]. Production of TNF and IL-1β is an early step in initiating skin inflammation, crucial for the recruitment of immune cells and subsequent amplification of the inflammatory cascade [22]. Dermal infiltration of immune cells and the inflammatory response may also impact the epidermal layer, leading to events such as epidermal hyperplasia [23,24]. 

Topical BaP application induced oxidative stress in epidermal cells, characterized by overwhelmed oxidative defense (as judged by a decreased GSH level), at both BaP doses, and increased mRNA expression of AhR and Cyp1A1 at a higher dose. Interaction of BaP with AhR could lead to the expression of Cyp1A1, metabolism of BaP and consequently oxidative stress [25]. At a lower BaP dose, a reduction in GSH level in epidermal cells, without alterations in AhR and Cyp1A1 mRNA expression, may be attributed to the activation of a more sensitive alternative pathway leading to the oxidative stress. This suggests the existence of pathways independent of AhR and Cyp1A1 that contribute to oxidative stress in a response to low-dose BaP exposure, as demonstrated in a previous study [26]. Analysis of epidermal cell immune response showed that, contrary to the skin explants, BaP decreased TNF and IL-1β responses. The decrease did not rely on BaP-induced cytotoxicity in the cell culture, as a similar reduction of MTT in control and treated cells was detected. These results are in disagreement with previous studies, which have shown that the in vitro response of keratinocytes to BaP is predominantly proinflammatory [10,27]. This discrepancy may be attributed to different model systems used (in vitro vs. in vivo), the dosage or the duration of the treatment. Next, we examined production of IL-10, an anti-inflammatory cytokine previously linked to cutaneous immunosuppression [28]. The increased IL-10 response by epidermal cells detected in this study could be associated with a decreased proinflammatory response by these cells, possibly as part of the regulatory mechanisms to dampen inflammation and promote tissue repair [29,30]. Beside keratinocytes, which are considered a primary source of IL-10 in murine epidermis, Langerhans cells (LC) and macrophages within this skin layer can also produce this cytokine [31]. BaP-induced immunosuppression mediated by IL-10 expression in an AhR-dependent manner has been previously shown for macrophages [32], but there is a lack of available data regarding the impact of BaP on the production of IL-10 in the skin. Despite decreased TNF and IL-1b responses in epidermal cells, their increase by skin explants could be attributed to cell types beyond the epidermal layer, such as fibroblasts, macrophages, neutrophils and mast cells [33]. This multifaceted response implies that BaP’s immunomodulatory effect on the skin is cell-specific, specifically in terms of localization within skin layers (epidermis or dermis). The reason for the differential skin response to BaP is not clear and deserves further investigation. 

Draining lymph nodes, along with the epidermal layer, play a crucial role in the initiation of the skin immune response. This study, for the first time, shows that topical exposure to BaP alone affects DLN cell response. The increased number of DLN cells following skin exposure to a higher BaP dose might be the result of DLN cell proliferation and/or an influx of various immune cells into the lymph nodes. Either process seems to involve non-T cells, as evidenced by the decreased relative number of both CD4^+^ and CD8^+^ cells. This decrease aligns with the reduced spontaneous production of IFN-γ and the production and mRNA expression of IL-17, cytokines typically linked with the functioning of effector CD4^+^ and CD8^+^ T cells. This suggests down-modulation of T-cell activity in DLN by BaP, and it is not associated with changes in the viability of DLN cells. Increased mRNA expression for IFN-γ, with concomitant decreased production of this cytokine, could indicate a post-transcriptional defect in IFN-γ, as previously demonstrated in peripheral blood mononuclear cells of children with atopic dermatitis [34]. Reduced Th1/type 1 and Th17/type 17 responses in DLN following BaP application seem not to be mediated by IL-10, as unchanged IL-10 spontaneous production and mRNA expression were detected. Additionally, despite this decrease it seems that T-cell function is not compromised, as DLN cells from BaP-treated animals still responded to strong mitogen ConA, which can directly stimulate T cells, by increasing both IFN-γ and IL-17 production. Concomitantly with increased IFN-γ and IL-17, ConA-stimulated production of IL-10 by DLN cells was increased, at least at a higher dose, which could indicate a host mechanism developed to prevent exaggerated T-cell activation [35]. BaP-induced reduction in the effector activity of DLN cells could be associated with immunosuppression detected in epidermal cells, as previously shown for dermatotoxic insults. A decreased proinflammatory response by epidermal cells, together with increased IL-10, could impair the function of skin dendritic cells (DC) or LC, leading to a suppression of the T-cell response in regional lymph nodes [36,37,38]. This assumption is in line with a previous study showing that dendritic cells, isolated from BaP-exposed skin, show abrogated stimulatory function when co-cultured with T cells [39].

In order to assess whether BaP’s multifaceted effect on cutaneous immune-mediated homeostasis alters the in vivo response to antigenic stimulus, we conducted skin sensitization with a contact hapten. This model is valuable for the analysis of the immunomodulatory effects of different chemicals/insults, especially those that gain access to the host system through the skin [40]. Following skin contact with haptens, an inflammatory signal develops in the skin, inducing migration of skin dendritic cells carrying uptaken hapten to DLN. This, in turn, leads to the activation of hapten-specific CD4^+^ and CD8^+^ T cells, typically assessed as a measure of skin sensitization [41]. Our previous studies showed that sensitization of DA rats with 0.4% DNCB resulted in the highest DLN response three days following sensitization, characterized by increased cell number and production of effector cytokines such as IFN-γ [42]. In this study, the suppressive effect of BaP on the immune response in DLN three days following skin sensitization was demonstrated, as evidenced by a decreased DLN number and the relative counts of CD4^+^ and CD8^+^ cells, together with decreased IFN-γ production. The decline in DLN cell number and effector activity was not associated with changes in DLN cell viability nor metabolic viability in culture between the groups. This suppression could be attributed to increased IL-10 production by DLN cells from BaP-treated sensitized animals, as previous studies showed that this cytokine impairs induction of CHS by suppressing the generation or function of effector T cells when different immunosuppressive treatments precede sensitization [31,43].

Although often underestimated as target tissue, skin is exposed to BaP in both occupational and environmental settings. Workers in aluminium smelters are considered to be exposed to highest level of PAH during a typical working life (40 years). Mid-range exposure is observed in workers employed in the road construction industry during asphalt mixing and road paving, and the lowest occupational exposure is seen in workers in coal processing industries, wood impregnation and power plants [44]. Large variations have been reported in the levels of occupational exposure between different studies, with maximum BaP values varying from <1 μg/m^3^ to 100 μg/ m^3^ in earlier studies to <0.001 μg/m^3^ to 26.2 μg/m^3^ in more recent data [45]. The highest environmental exposure to BaP is reported to be seen in urban cities with heavy traffic, industrial areas and cigarette-polluted areas [46]. Thanks to its lipophilicity, after deposition on the skin, BaP dissolves in lipid-rich stratum corneum. There, it can make strong interactions and form reservoirs, further affecting barrier function and diffusing into other skin layers [2,10]. Previous studies have shown that BaP, among other PAHs, has the greatest accumulation level in skin following topical application from an aqueous medium [10], where it can affect keratinocytes and skin immune activity. Considering epidermal cells as key immune sentinels for skin defense, the BaP-induced decrease in epidermal cell immune function, along with reduced IFN-γ and IL-17 response in DLN, detected in this study suggests a potential impairment in skin immunosurveillance against tumors or infectious agents, but in vaccination efficacy as well [37]. Although the link between dermal exposure to BaP and increased occurrence of skin tumors is established [47], the potential effects of BaP on skin susceptibility to infections or on vaccination efficiency remain largely unexplored. These findings highlight the need for future research to elucidate these impacts in order to better understand how environmental and occupational BaP exposure could affect skin health and pose a risk for overall health. Moreover, given that the skin is exposed to BaP as part of PAHs mixture in environmental and occupational settings, and considering that PAH mixtures increase skin permeability and alter metabolism compared to single BaP [2], which potentially could affect skin immunotoxicity [10,48], awareness should be raised of the cumulative effects of PAH mixtures on skin immune responses. This underscores the need for further research to properly estimate the health risk of real-life skin exposure to complex PAH mixtures.

## 4. Material and Methods

### 4.1. Chemicals

Benzo[a]pyrene (BaP), Concanavalin A (ConA), Dispase II and 3-(4, 5-dimethyl-thiazol-2-yl)-2, 5 diphenyltetrazolium bromide (MTT) were purchased from Sigma-Aldrich (Sigma Chemical Co., St. Louis, MO, USA). Trypsin solution was obtained by Difco (Lawrence, KS, USA) and glutathione (GSH) from Fluka Chemie (Buchs, Switzerland). Fluoroisothiocyanate (FITC)-labeled mouse antibodies to rat CD4^+^ and phycoerythrin (PE)-labeled mouse antibodies to rat CD8^+^ were purchased from eBioscience (eBioscience Inc., San Diego, CA, USA). Culture medium RPMI-1640 (Corning, NY, USA) supplemented with 2 mM glutamine, 20 μg/mL gentamycine (Galenika a.d, Zemun, Serbia) and 5% (*v*/*v*) heat inactivated FCS (fetal calf serum) (Corning, NY, USA) were used. For use in experiments, ConA and dispase II were dissolved in RPMI-1640 medium. BaP was dissolved in dimethyl sulfoxide (DMSO) (ChemCruz, Santa Cruz Biotechnology, Inc., Santa Cruz, CA, USA). All solutions for cell culture experiments were prepared under sterile conditions and were sterile-filtered (Minisart, pore size 0.20 mm, Sartorius Stedim Biotech, Goettingen, Germany) before use. One-chloro-2,4-dinitrobenzene (DNCB) was obtained from BDH Chemicals Ltd., Hull, UK and dissolved in 4:1 acetone:olive oil. 

### 4.2. Animals and Treatment with Benzo[a]pyrene (BaP) 

The experimental part of the research and the treatment of animals were realized according to EEC Directive (86/609/EEC) on the protection of animals used for experimental and other scientific purposes, with the approval of the Veterinary Directorate, Ministry of Agriculture, Forestry and Water Management, Republic of Serbia (ethical clearance number: 323-07-10703/2022-05). Eight- to ten-week-old male rats of the dark agouti (DA) strain, conventionally housed in IBISS, in a controlled environment (12 h light:dark cycle, a 60% relative humidity and 21–24 °C temperature), were used in the experiments. The experiments were repeated two or three times and within each treatment the animals were divided into three groups with 4–5 animals per group. BaP, dissolved in DMSO, was diluted in phosphate-buffered saline (PBS) to final concentrations of 2.4 µg/mL and 12 µg/mL (working concentrations). Animals were treated with 100 µL of BaP working concentrations for 10 consecutive days to the upper part of the dorsum (12 cm^2^), clipped of fur, to achieve a daily concentration of 20 ng/cm^2^ and 100 ng/cm^2^. These doses are notably lower compared to those used in other animal studies involving topical application of BaP [49,50], aligning more closely with levels of everyday or occupational exposure [4,51]. Specifically, the dose of 20 ng/cm^2^ used in this study corresponds approximately to airborne exposure of 200 ng/m^3^ [52], mimicking environmental exposure in high pollution settings (urban cities with heavy traffic, industrial areas, cigarette-polluted areas). The five-times-higher dose of 100 ng/cm^2^ rather corresponds to low occupational exposure, reaching 1 μg BaP/m^3^ (coal liquefaction, wood impregnation, power plants) [45]. Control groups of rats were treated with PBS/0.25% DMSO on the same skin area. Twenty-four hours following the last BaP application, skin and DLN were collected.

During the study, all rats had ad libitum access to water and standard rodent pellets. After the treatment, the animals were anesthetized by IP injection of 15 mg/kg b.w. of Zoletil 100 (Virbac, Carros, France). There were no differences in body mass increase during the experiments between the groups and no differences in body mass at the end of the experiment (158.00 ± 14.54 g in controls, 165.67 ± 13.94 g at the lower dose and 163.40 ± 23. 87 g at the higher dose).

### 4.3. Histology 

Sections of the full-thickness treated skin were fixed in 4% formaldehyde (pH 6.9). The tissues underwent dehydration through a series of increasing alcohol concentrations, followed by a rinse in xylene. After washing, the skin parts were embedded in paraffin and sectioned at 5 μm. The histological preparations were stained with hematoxylin and eosin and analyzed using a Coolscope digital light microscope (Nikon, Tokyo, Japan) by a certified histologist. Additionally, histological sections were stained with Giemsa stain in order to evaluate the number of mast cells. 

### 4.4. Culture of Skin Explants

Skin samples, cleared of subcutaneous tissue, were cut into pieces of approximately 1 cm^2^ and placed in 1 mL of complete culture medium. Skin explants were incubated for 48 h in culture medium, followed by centrifugation and collection of supernatant (conditioned medium), where the levels of cytokines were determined.

### 4.5. Isolation and Culture of Epidermal Cells and Draining Lymph Nodes

Treated skin was cut into small pieces and incubated overnight with Dispase II (2.5 mg/mL) at 4 °C. After overnight incubation, the epidermis was separated from the dermis and epidermal layers were incubated with trypsin–glucose solution (0.25% trypsin, 0.1% glucose) for 30 min at 37 °C. Cells from lymph nodes (axillar and suprascapular), that drain the treated part of the skin, were obtained after mechanical extrusion through nylon mesh (70 μm nylon, BD Bioscience, Bedford, MA, USA). Epidermal and DLN cells were counted by improved Neubauer hemocytometer, and cell viability was determined by trypan blue exclusion assay. For determination of cytokine production, isolated epidermal cells (0.2 × 10^6^ cells/well) or DLN cells (1.2 × 10^6^ cells/well) were cultured in 96-well plates for 48 h, after which culture supernatant (conditioned medium) was collected by centrifugation. To determine cell metabolic viability, 100 µL of medium containing 10 µL of MTT salt was added immediately to freshly isolated cells or after 48 h of culture. The cells were then incubated for 3 h at 37 °C in a humidified atmosphere containing 5% CO_2_. At the end of the incubation, 10% sodium dodecyl sulfate (SDS)–0.01 N HCl was added to dissolve the formed formazan, and the absorbance of extracted chromogen was read spectrophotometrically at 540 nm. 

### 4.6. ELISA

Concentrations of rat TNF (Invitrogen, Carlsbad, CA, USA) and IL-1β (R&D systems, Minneapolis, MN, USA) were determined in the supernatant of cultured epidermal cells and skin explants. In the supernatant of cultured DLN cells, mouse IL-17 cross-reactive with rat IL-17 (Invitrogen, USA), IFN-γ and IL-10 (purchased from R&D, Minneapolis, MN, USA) were measured. From the constructed standard curve with known amounts of recombinant cytokines, the concentrations of the investigated cytokines were obtained. Concentrations were expressed in pg per mL.

### 4.7. Determination of GSH Levels in Epidermal Cells 

Isolated epidermal cells (1 × 10^6^) were lysed in a 10 mM HCl for determination of GSH level. Following protein precipitation with 5% sulphosalicylic acid, GSH in the supernatant was measured spectrophotometrically at 412 nm using DTNB in Tris–HCl (pH 8.9). A standard curve was constructed using reduced glutathione, and the results were expressed as μmol of GSH/mg of proteins.

### 4.8. Reverse Transcription Real-Time Polymerase Chain Reaction (RT-PCR) 

RNA (1 μg) was isolated from epidermal cells and DLN cells using an mi-Total RNA Isolation Kit (Metabion, Martinsried, Germany) and reverse transcribed using random hexamer primers and MMLV (Moloney Murine Leukemia Virus) reverse transcriptase (Fermentas, Vilnius, Lithuania), following the manufacturer’s instructions. Gene expression of AhR, Cyp1A1, IL-1β and TNF was determined in epidermal cells, while gene expression of IL-17, IFN-γ and IL-10 was measured in the DLN cells. According to recommendations, prepared cDNAs were amplified using Power SYBR^®^ Green PCR Master Mix (Applied Biosystems, Foster City, CA, USA) in a total volume of 20 μL in Quant StudioTM 3 Real-Time PCR Instrument (96-well 0.2-mL) (Applied Biosystems). Thermocycler conditions were: an initial step at 50 °C for 5 min, followed by a step at 95 °C for 10 min and subsequent 2-step PCR program at 95 °C for 15 s and 60 °C for 60 s for 40 cycles. PCR primers (forward/reverse) used in the study were:

AhR: 5′-GCTGTGATGCCAAAGGGCAGC-3′/5′-TGAAGCATGTCAGCGGCGTGGAT-3′; 

Cyp1A1: 5′-GGGGAGGTTACTGGTTCTGG-3′/5′-CGGATGTGGCCCTTCTCAAA-3′;

IL-1β: 5′-CACCTCTCAAGCAGAGCA-3′/5′-GGGTTCCATGGTGAAGTCAAC-3′; 

TNF: 5′-TCGAGTGACAAGCCCGTAGC3′/5′-CTCAGCCACTCCAGCTGCTC-3′ 

IFN-γ: 5′-TGGCATAGATGTGGAAGAAAAGAG-3′/5′-TGCAGGATTTTCATGTCACCAT-3′

IL-17:5′-ATCAGGACGCGCAAACATG-3′/5′-TGATCGCTGCTGCCTTCAC-3′

β-actin (housekeeping reporter gene): 5′-CCCTGGCTCCTAGCACCAT-3′/5′GAGCCACCAATCCACACAGA-3′.

PCR products were detected in real time, and the results were analyzed using Quant StudioTM Design & Analysis Software v1.4.3 (Applied Biosystems). Results were calculated as 2-dCt, where dCt represented the difference between the threshold cycle (Ct) values of a specific gene and the endogenous control (β-actin).

### 4.9. Skin Sensitization with DNCB

Experimental contact allergen DNCB, dissolved in acetone/olive oil in 4:1 ratio, was used for induction of animal sensitization. After 10 days of treatment with 100 μL of PBS/0.25% DMSO (control group) and BaP 100 ng/cm^2^, 100 μL of 0.4% DNCB was applied to the same treatment area over the next two days in order to induce skin sensitization [42]. Three days (72 h) following the second DNCB application, DLN were isolated and all functional measurements were carried out. Animals were anesthetized by IP injection of 15 mg/kg b.w. of Zoletil 100 (Virbac, Carros, France). 

### 4.10. Flow Cytometry 

Samples of DLN cells whose concentration was 1 × 10^6^ were incubated on ice for 30 min with FITC-conjugated antibodies to rat CD4^+^ and PE-labeled antibodies for rat CD8^+^ cells. After washing twice with PBS, the cells were fixed with 1% paraformaldehyde (Serva, Heidelberg, Germany) and kept in the dark at 4 °C until analysis. Fluorescence intensity was analyzed by CytoFLEX flow cytometer (Beckman Coulter, Indianapolis, IN, USA).

### 4.11. Statistical Analysis 

All data are presented as mean (±standard deviation) from two or three independent experiments. Statistical analysis was performed using STATISTICA 7.0 (StatSoft Inc., Tulsa, OK, USA). For multiple group comparison, the Kruskal–Wallis test, followed by a multiple comparison per mean rank sum post hoc test, was used. For comparison between two groups, the Mann–Whitney test was used. *p*-values less than 0.05 were considered significant.

## 5. Conclusions

The findings of this study reveal the complex immunomodulatory effect of topically applied BaP in environmentally and occupationally relevant doses. BaP-induced epidermal damage and dermal infiltration were associated with an increased proinflammatory response by the skin explants. Concurrently, BaP induced a local immunosuppression characterized by a decreased proinflammatory response of epidermal cells associated with an increased anti-inflammatory IL-10 response, together with reduced Th1/type 1 and type 17/Th17 responses in DLN. This immunosuppression seems to impair the skin’s immune response to experimental contact haptens. These results provide new insight into the immunomodulatory effects and health risks associated with skin exposure to BaP.

## Figures and Tables

**Table 1:** Draining lymph node cellularity, cell viability and basic phenotypic analysis following BaP application.

	Bap Dose (ng/cm^2^)		
	0	20	100
Cell number (×10^6^)	13.08 ± 2.30	13.11 ± 6.11	17. 66 ± 6.08 *
Cell viability (%)	93.10 ± 2.21	92.14 ± 1.98	94.78 ± 1.30
MTT reduction (A_540nm_)	0.23 ± 0.09	0.23 ± 0.05	0.26 ± 0.05
CD4^+^ (%)	57.49 ± 2.01	54.84 ± 3.94	51.83 ± 3.90 *
CD8^+^ (%)	17.53 ± 1.26	14.46 ± 1.91	13.17 ± 0.28 *

Results are presented as mean (±SD). Significance at: * *p* < 0.05 vs. control animals (BaP dose 0 ng/cm^2^).

**Table 2:** Effect of epicutaneous BaP application on DLN cells response following skin sensitization with DNCB.

	Sensitization with 0.4% DNCB	
	BaP (ng/cm^2^)	
	0	100
Cell number (×10^6^)	53.50 ± 8.53	41.80 ± 6.64 *
Cell viability (%)	91.89 ± 0.63	92.45 ± 0.64
MTT reduction (A_540nm_)	0.30 ± 0.03	0.42 ± 0.02 *
CD4^+^ (%)	59.44 ± 0.92	56.81 ± 1.21 *
CD8^+^ (%)	24.39 ± 0.88	22.37 ± 0.54 **
IFN-γ (pg/mL)	73.06 ± 12.76	30.82 ±15.38 **
IL-10 (pg/mL)	113.13 ± 8.50	149.00 ± 27.50 *

Results are presented as mean (±SD). Significance at: * *p* < 0.05, ** *p* < 0.01 vs. control animals (BaP dose 0 ng/cm^2^).

## Data Availability

Data is contained within the article or Supplementary Materials.

## Supplementary Materials

The following are available online at https://www.mdpi.com/article/10.3390/ijms25168631/s1. Original microscopy images, used for preparation of Figure 1, are provided as Supplementary Data S1.
