# Supplementary figures and images for: Impact of Skin Exposure to Benzo[a]pyrene in Rat Model: Insights into Epidermal Cell Function and Draining Lymph Node Cell Response
Source: Int J Mol Sci. 2024 Aug 8;25(16):8631. doi: 10.3390/ijms25168631 (PMC11354278; doi:10.3390/ijms25168631)

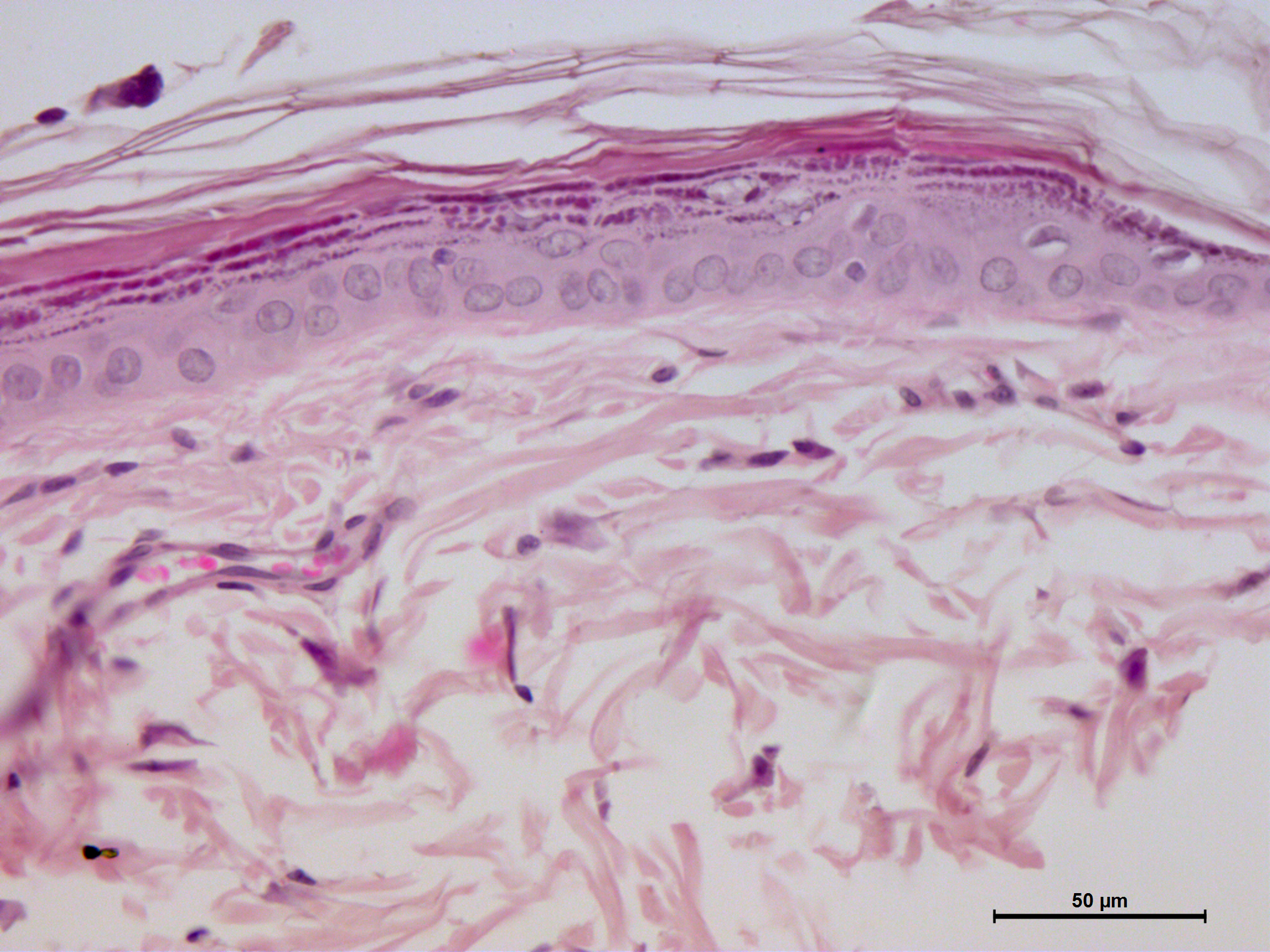

Supplement: Supplementary file 1 [file ijms-25-08631-s001.zip › Figure 1A (insert).tif]

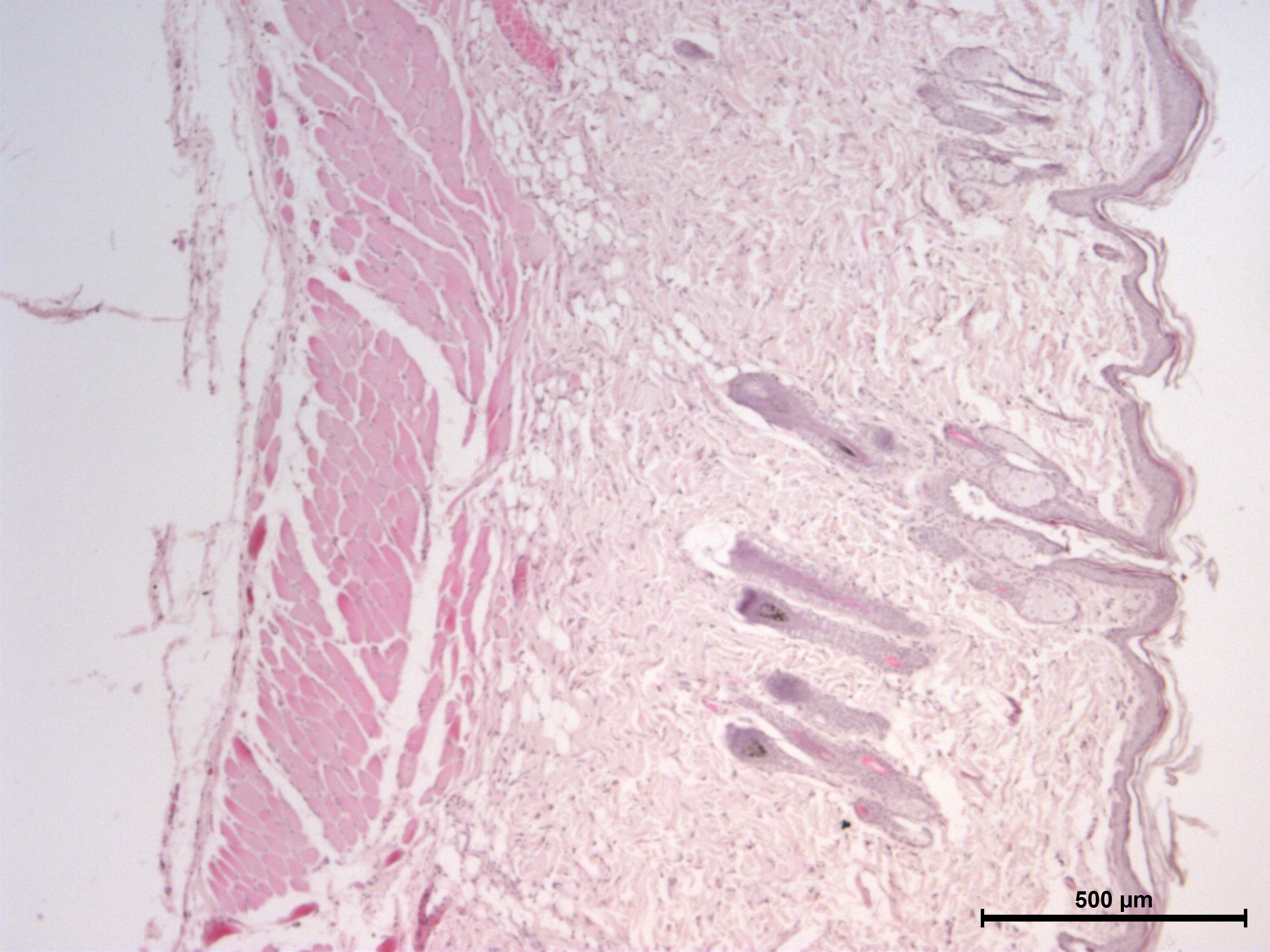

Supplement: Supplementary file 1 [file ijms-25-08631-s001.zip › Figure 1A.tif]

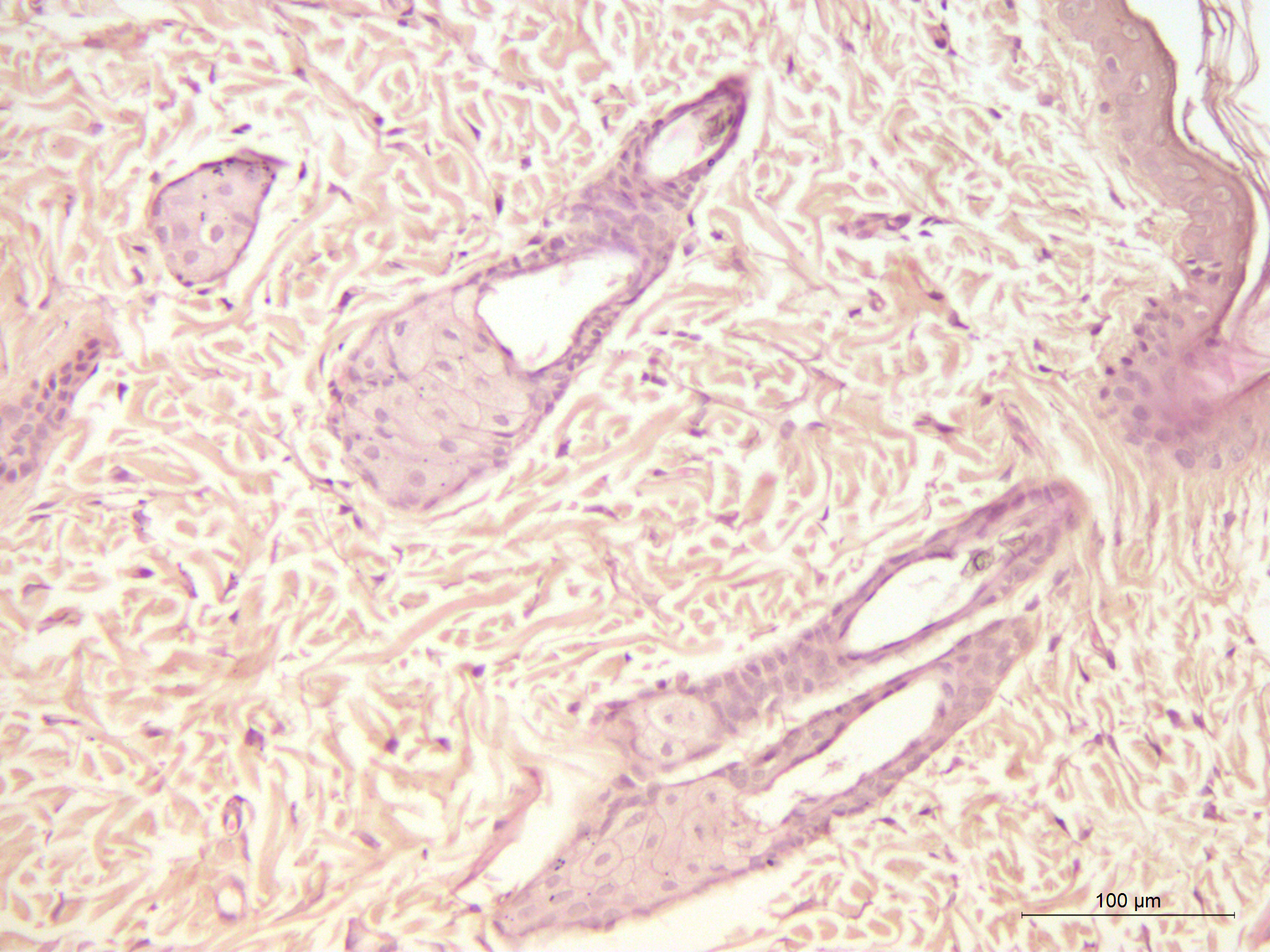

Supplement: Supplementary file 1 [file ijms-25-08631-s001.zip › Figure 1B.tif]

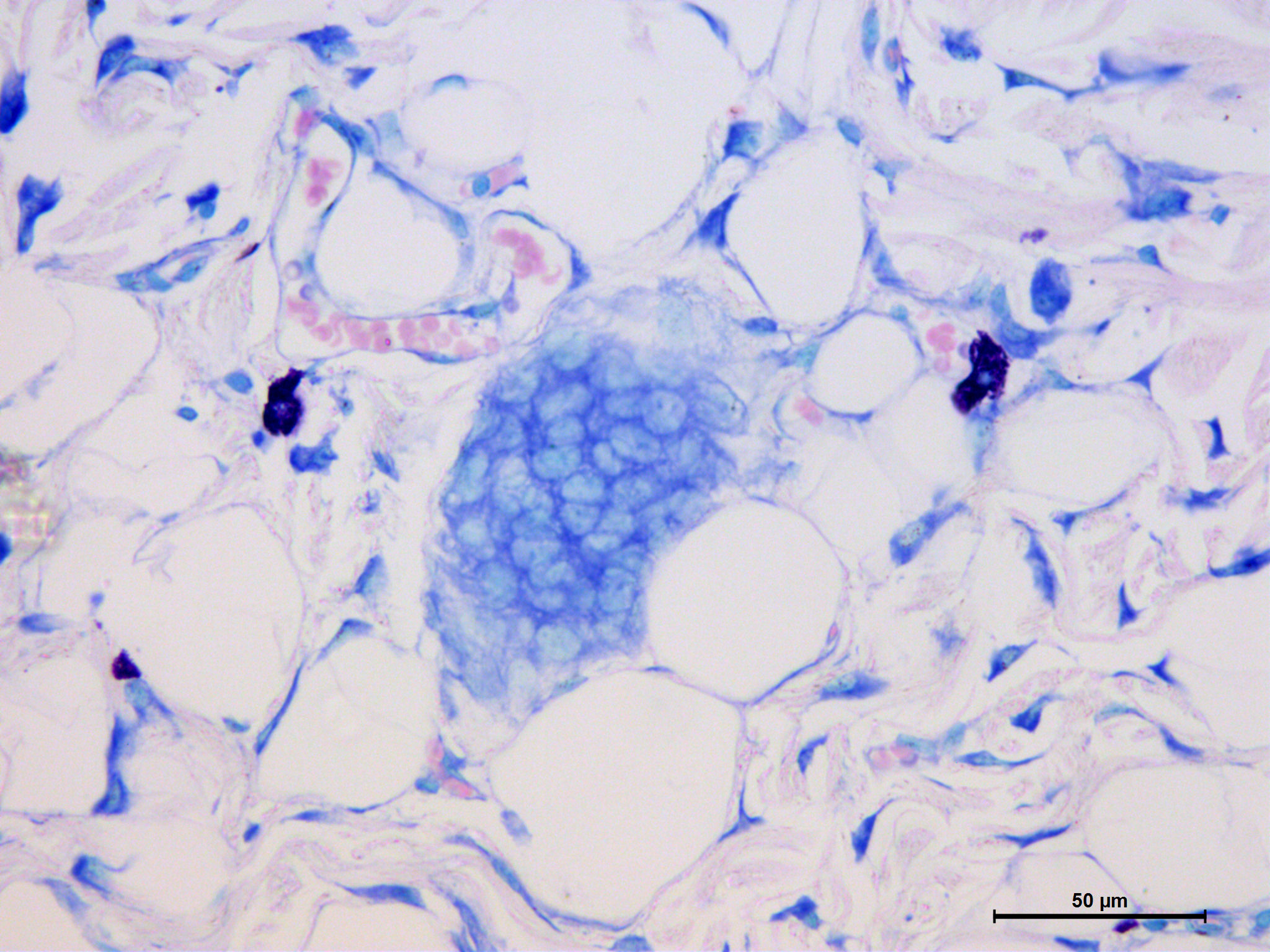

Supplement: Supplementary file 1 [file ijms-25-08631-s001.zip › Figure 1C (insert).tif]

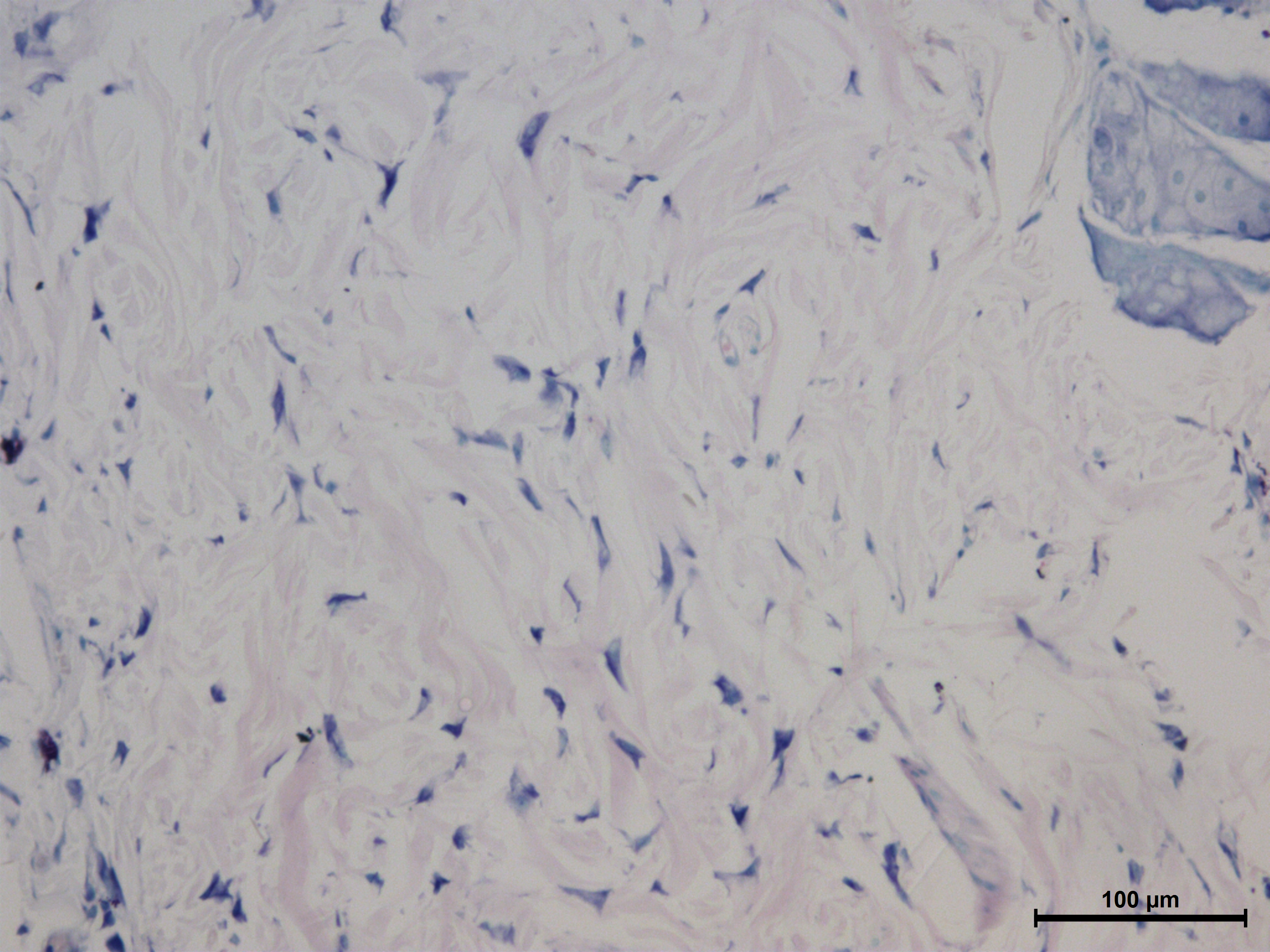

Supplement: Supplementary file 1 [file ijms-25-08631-s001.zip › Figure 1C.tif]

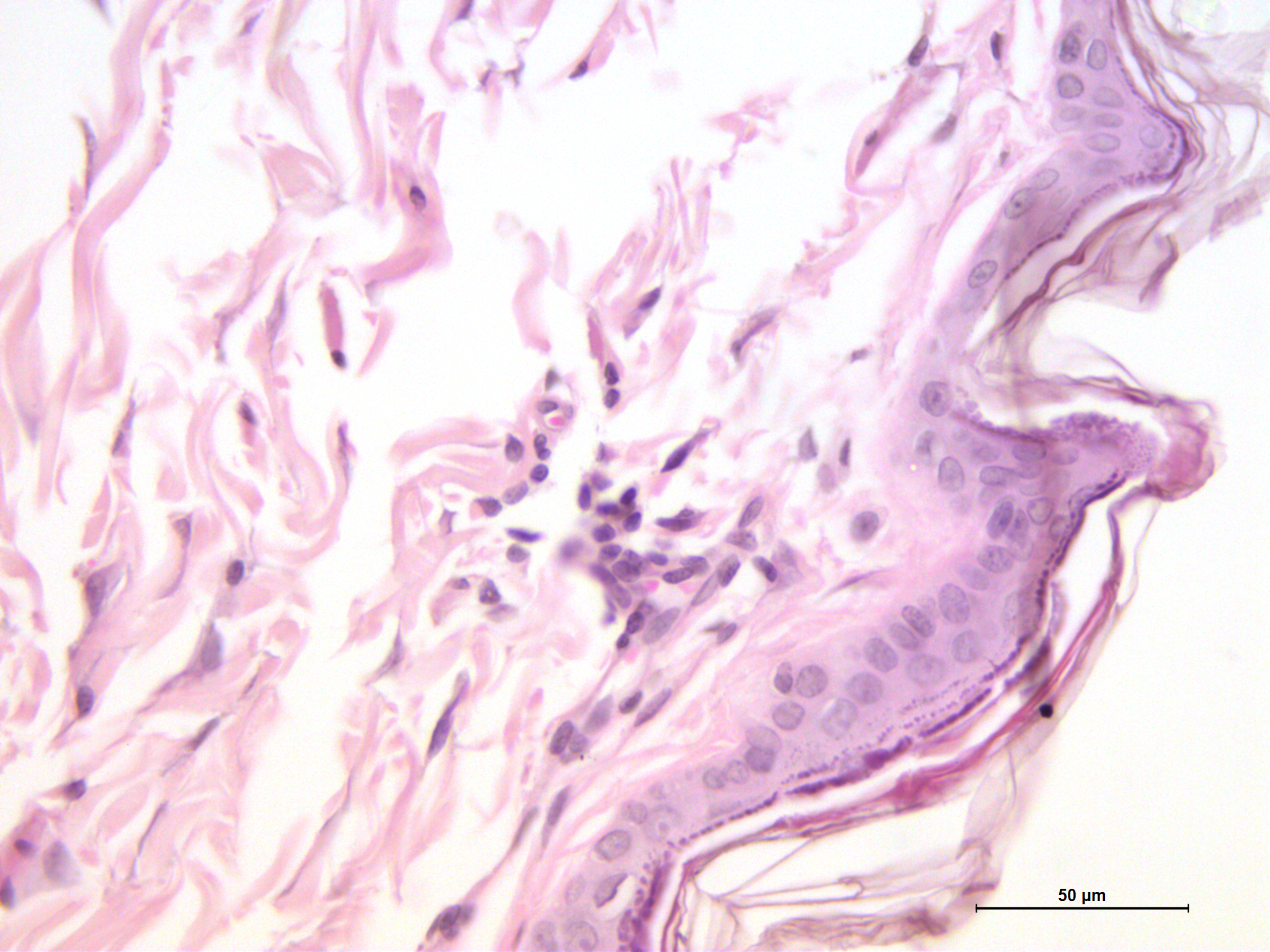

Supplement: Supplementary file 1 [file ijms-25-08631-s001.zip › Figure 1D (bottom insert).tif]

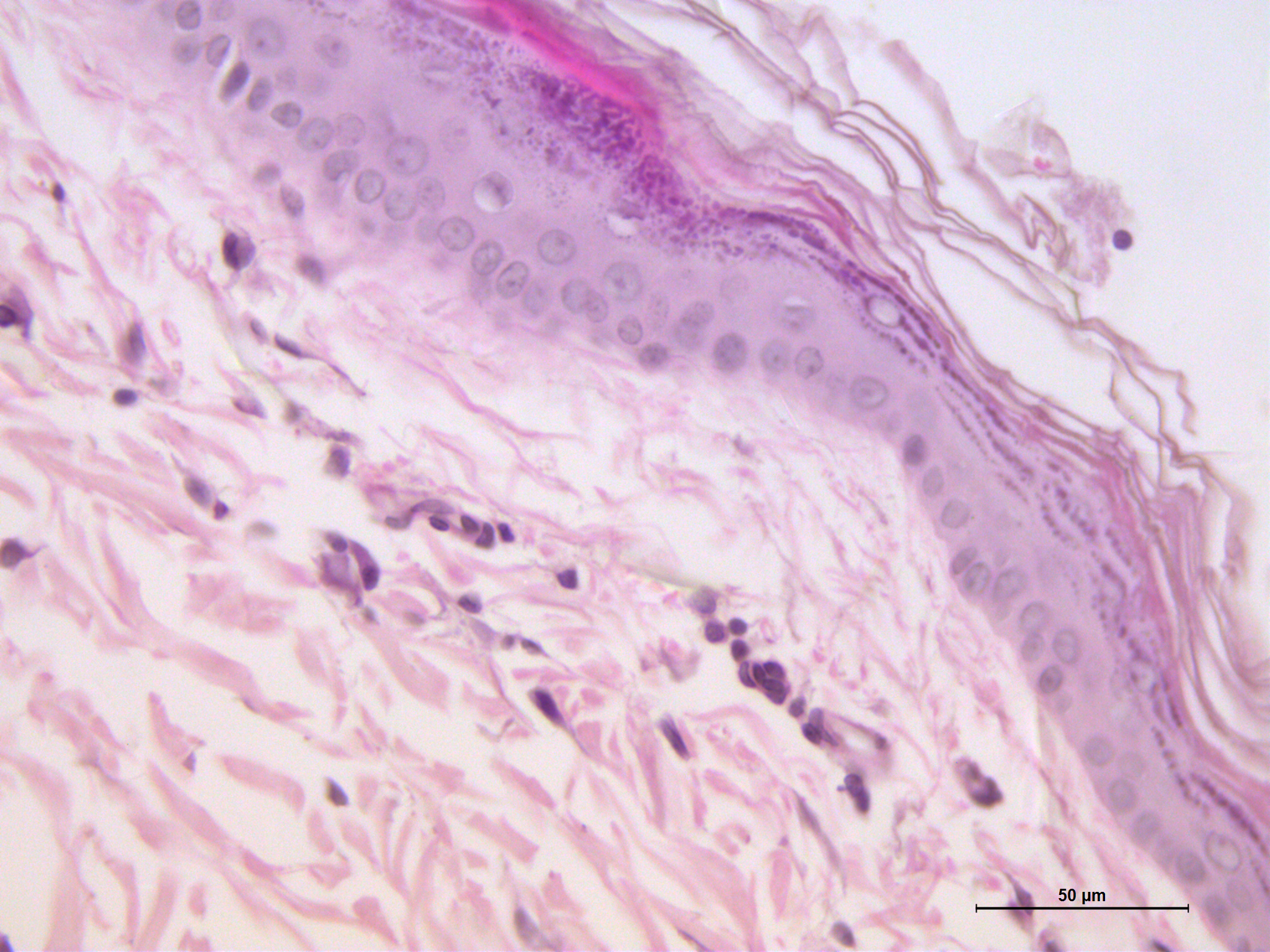

Supplement: Supplementary file 1 [file ijms-25-08631-s001.zip › Figure 1D (top insert).tif]

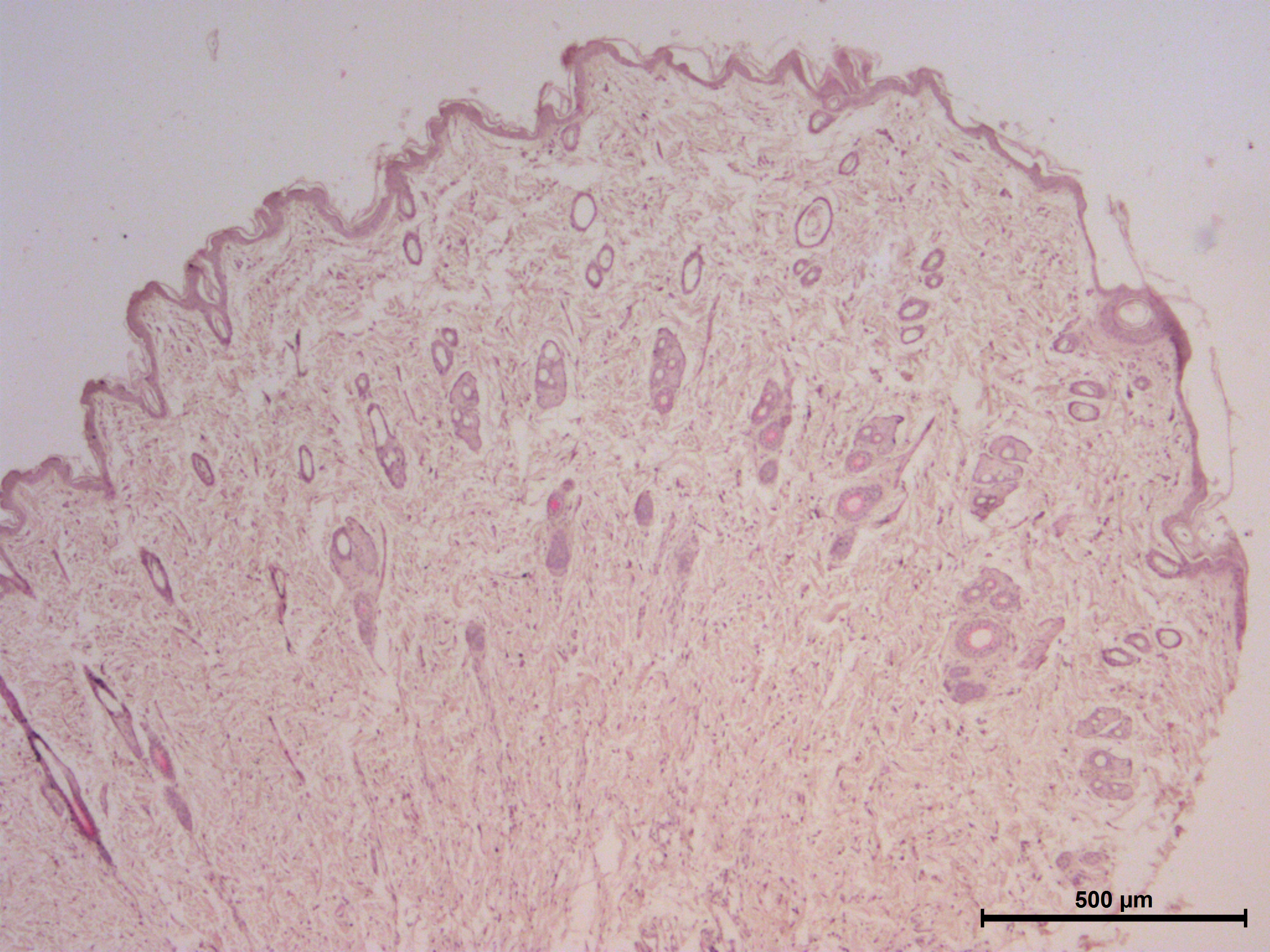

Supplement: Supplementary file 1 [file ijms-25-08631-s001.zip › Figure 1D.tif]

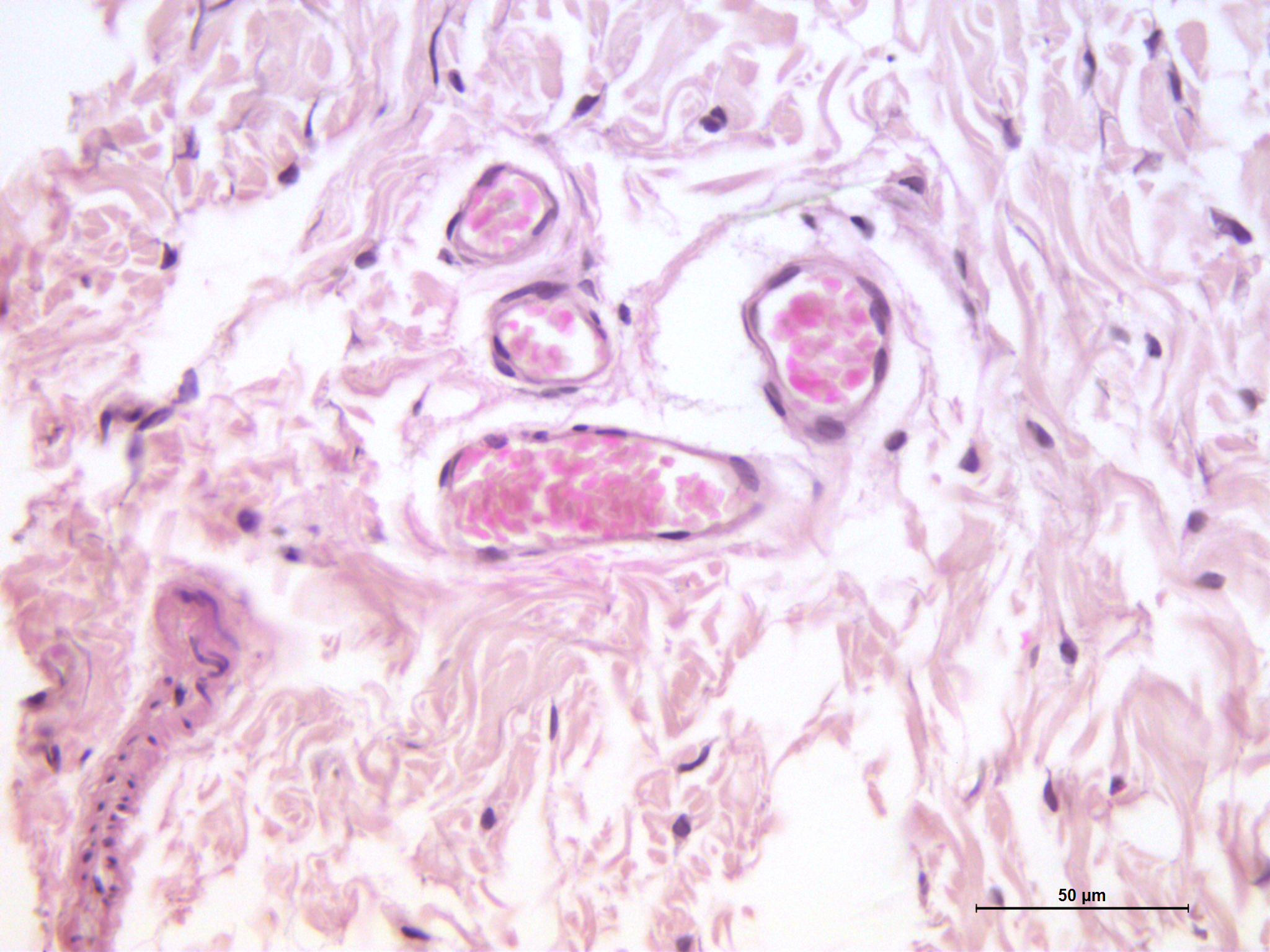

Supplement: Supplementary file 1 [file ijms-25-08631-s001.zip › Figure 1E (insert).tif]

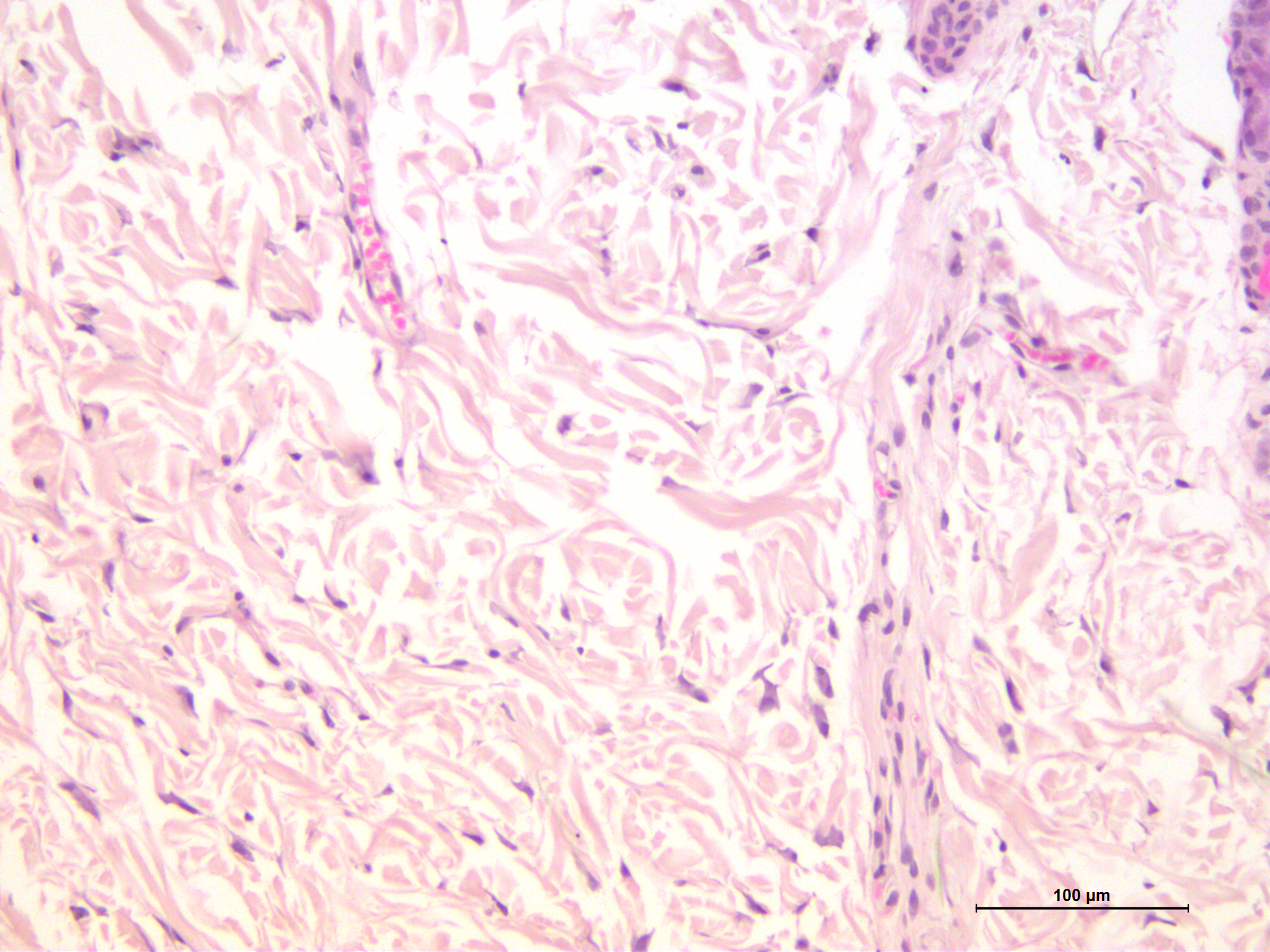

Supplement: Supplementary file 1 [file ijms-25-08631-s001.zip › Figure 1E.tif]

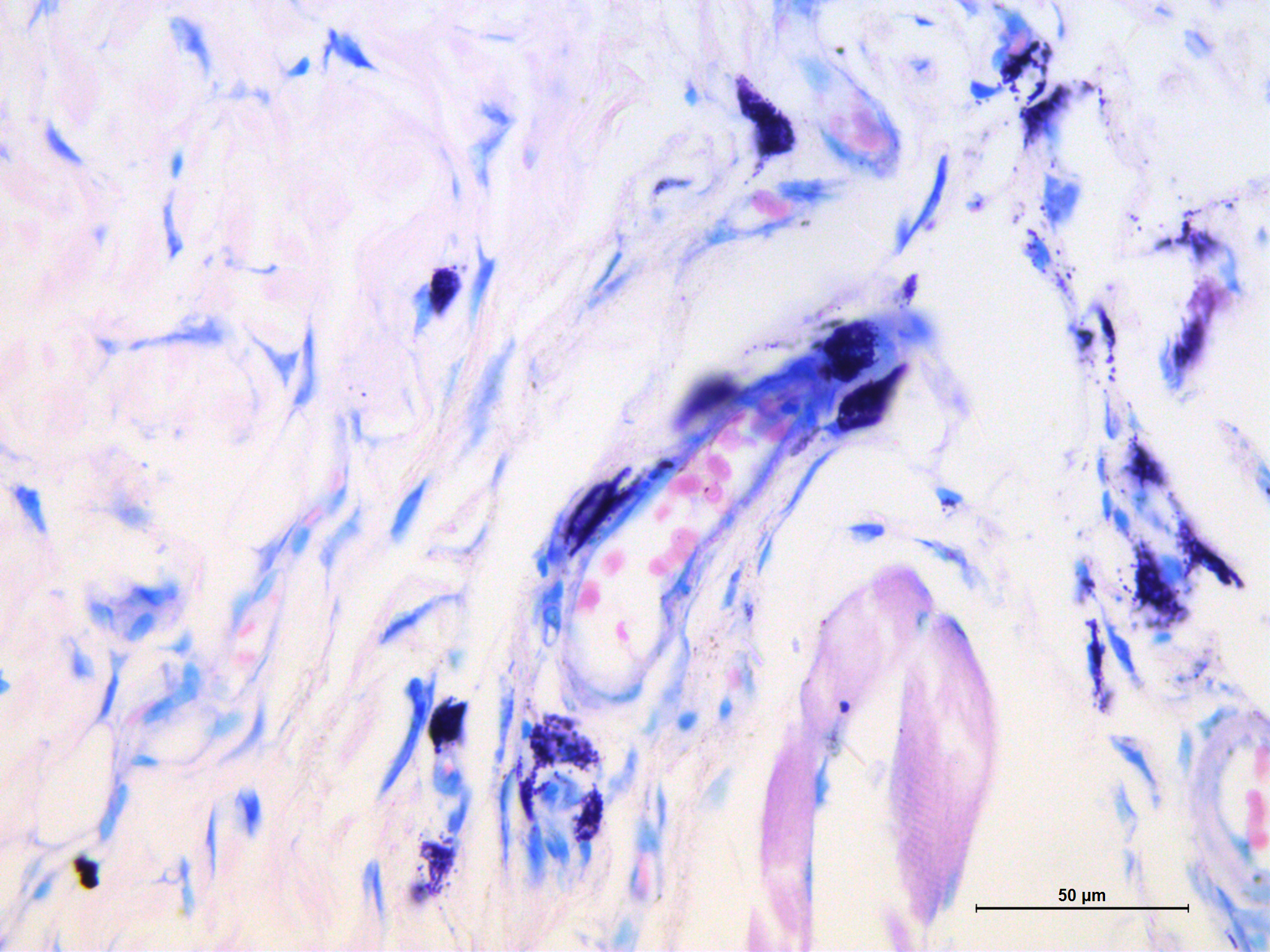

Supplement: Supplementary file 1 [file ijms-25-08631-s001.zip › Figure 1F (insert).tif]

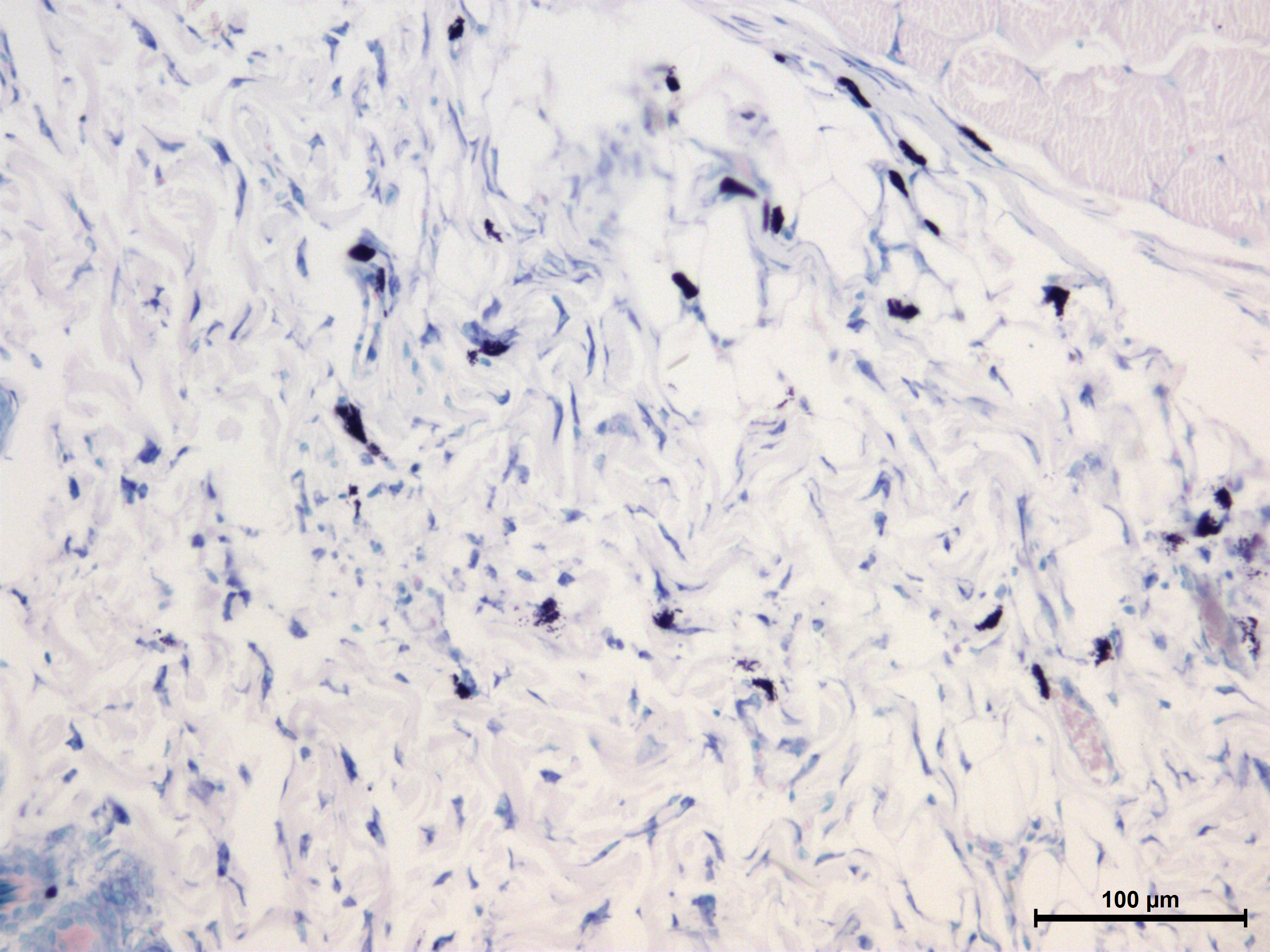

Supplement: Supplementary file 1 [file ijms-25-08631-s001.zip › Figure 1F.tif]

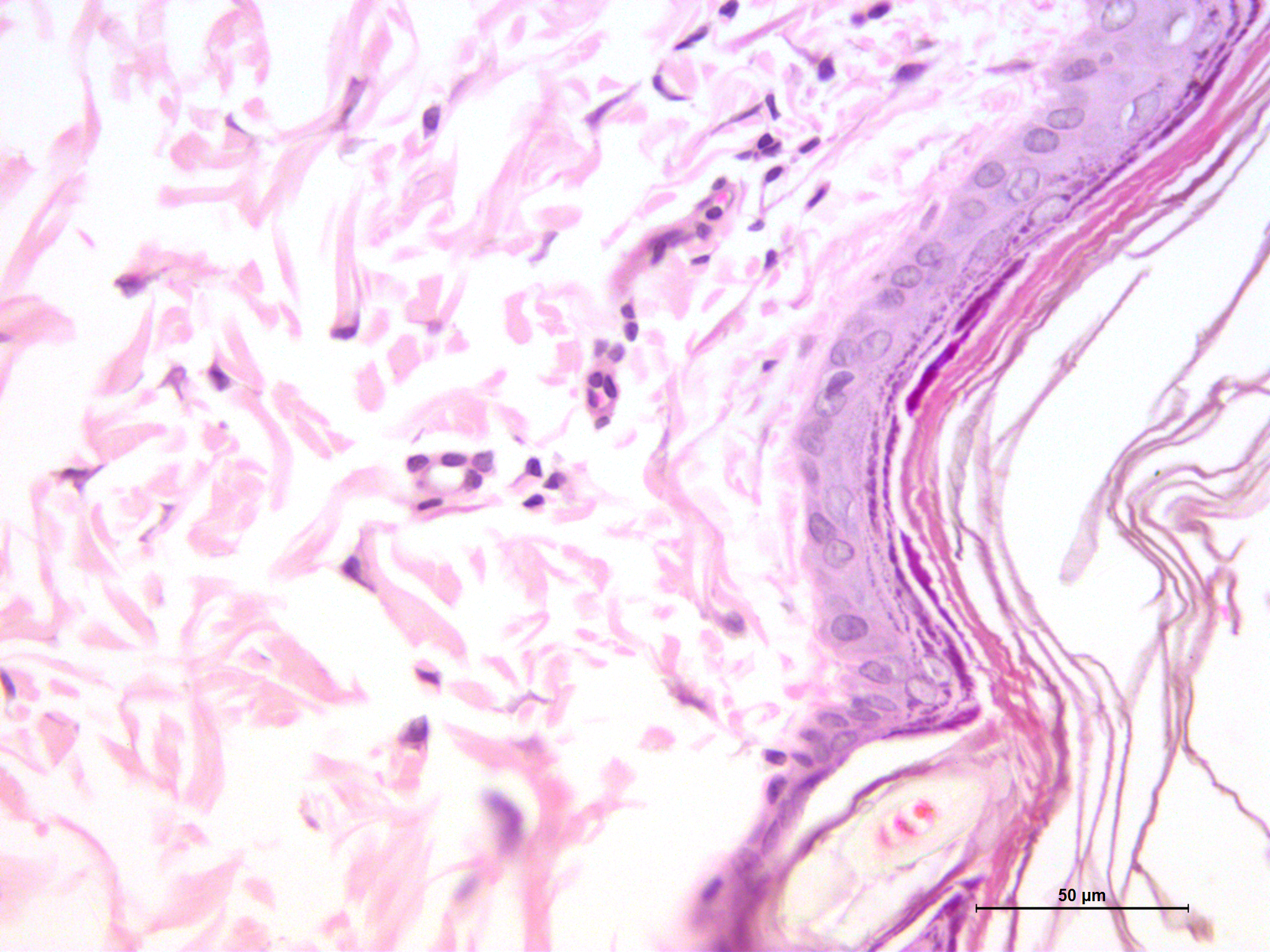

Supplement: Supplementary file 1 [file ijms-25-08631-s001.zip › Figure 1G (bottom insert).tif]

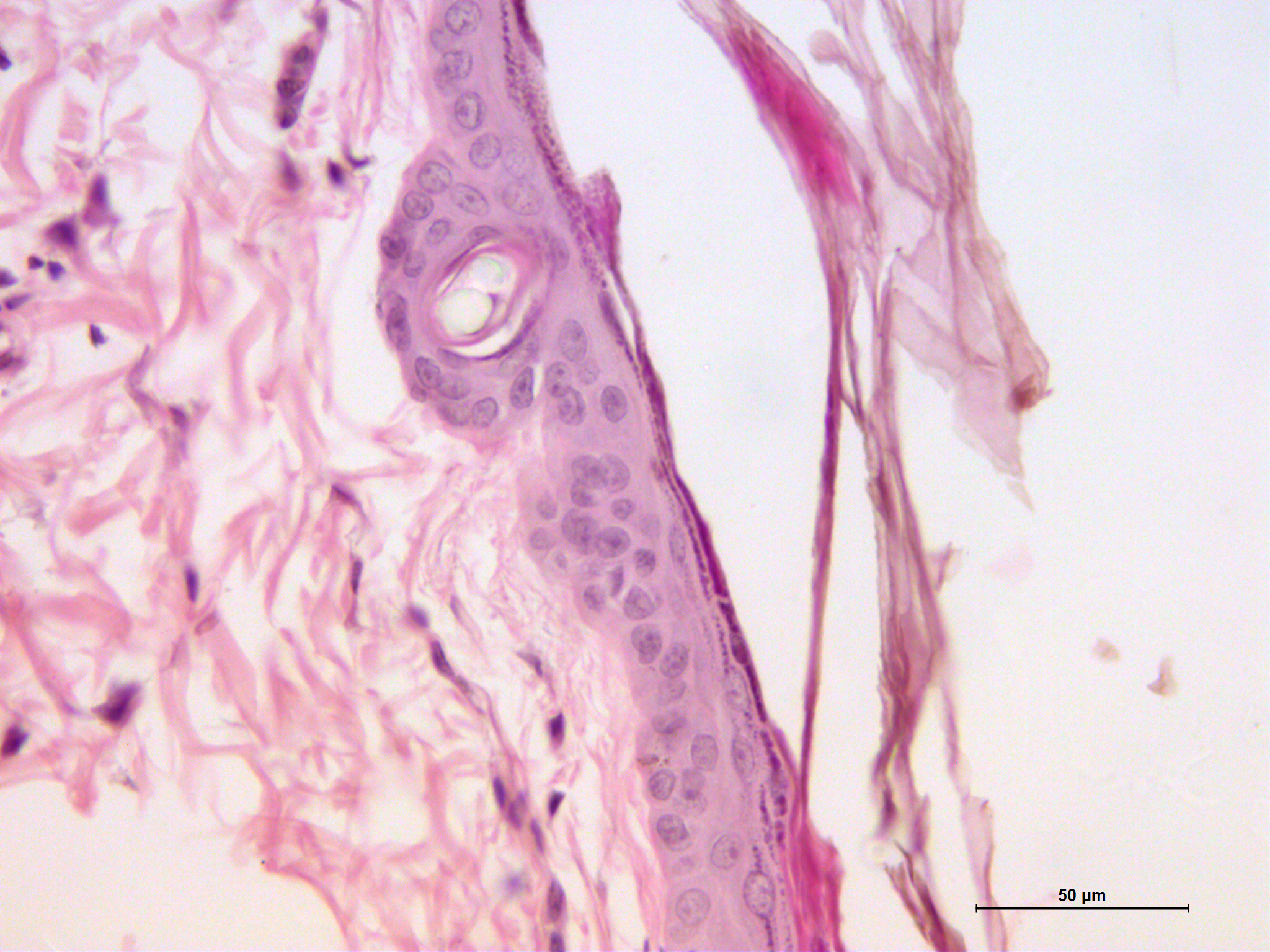

Supplement: Supplementary file 1 [file ijms-25-08631-s001.zip › Figure 1G (top insert).tif]

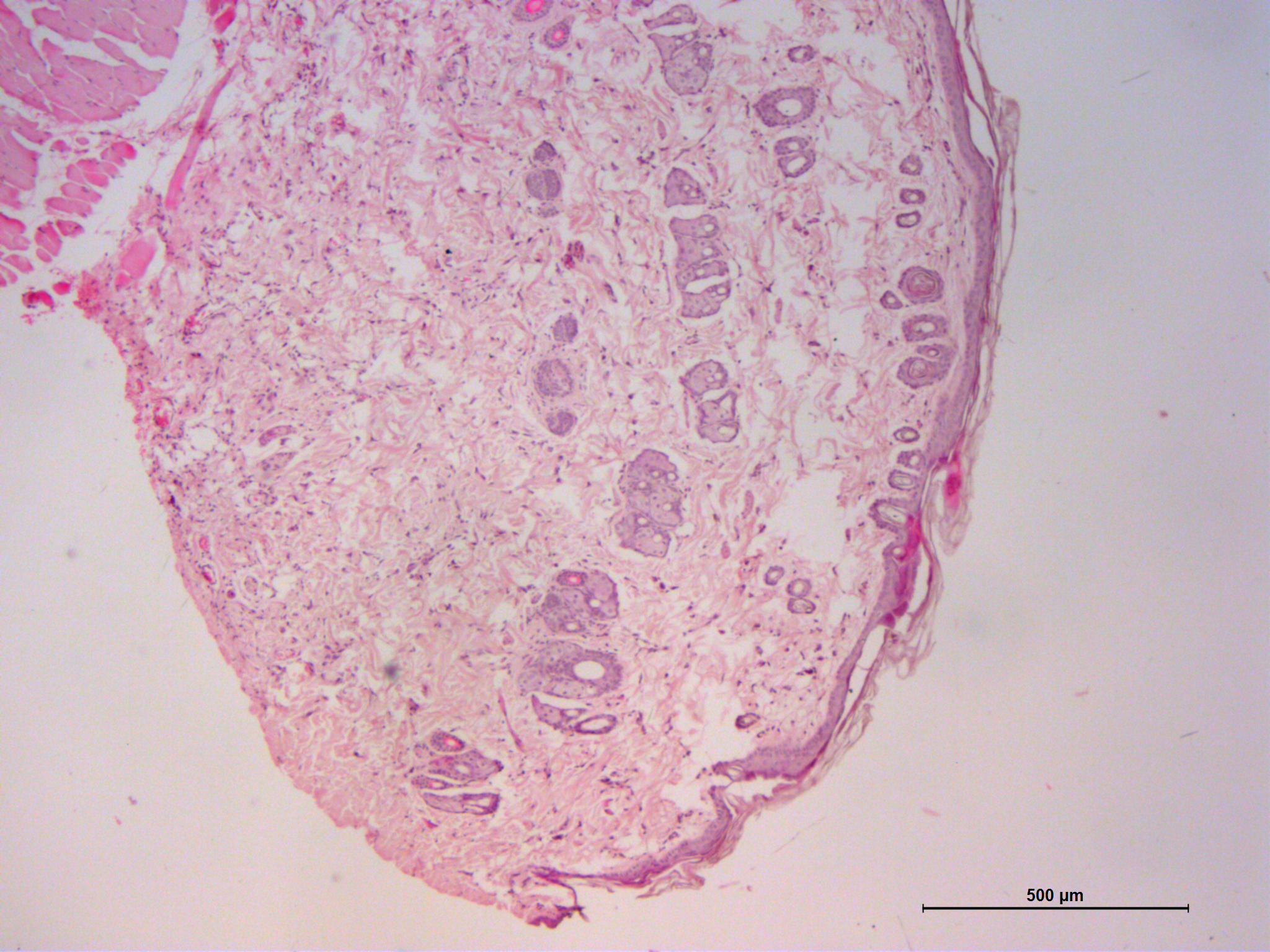

Supplement: Supplementary file 1 [file ijms-25-08631-s001.zip › Figure 1G.tif]

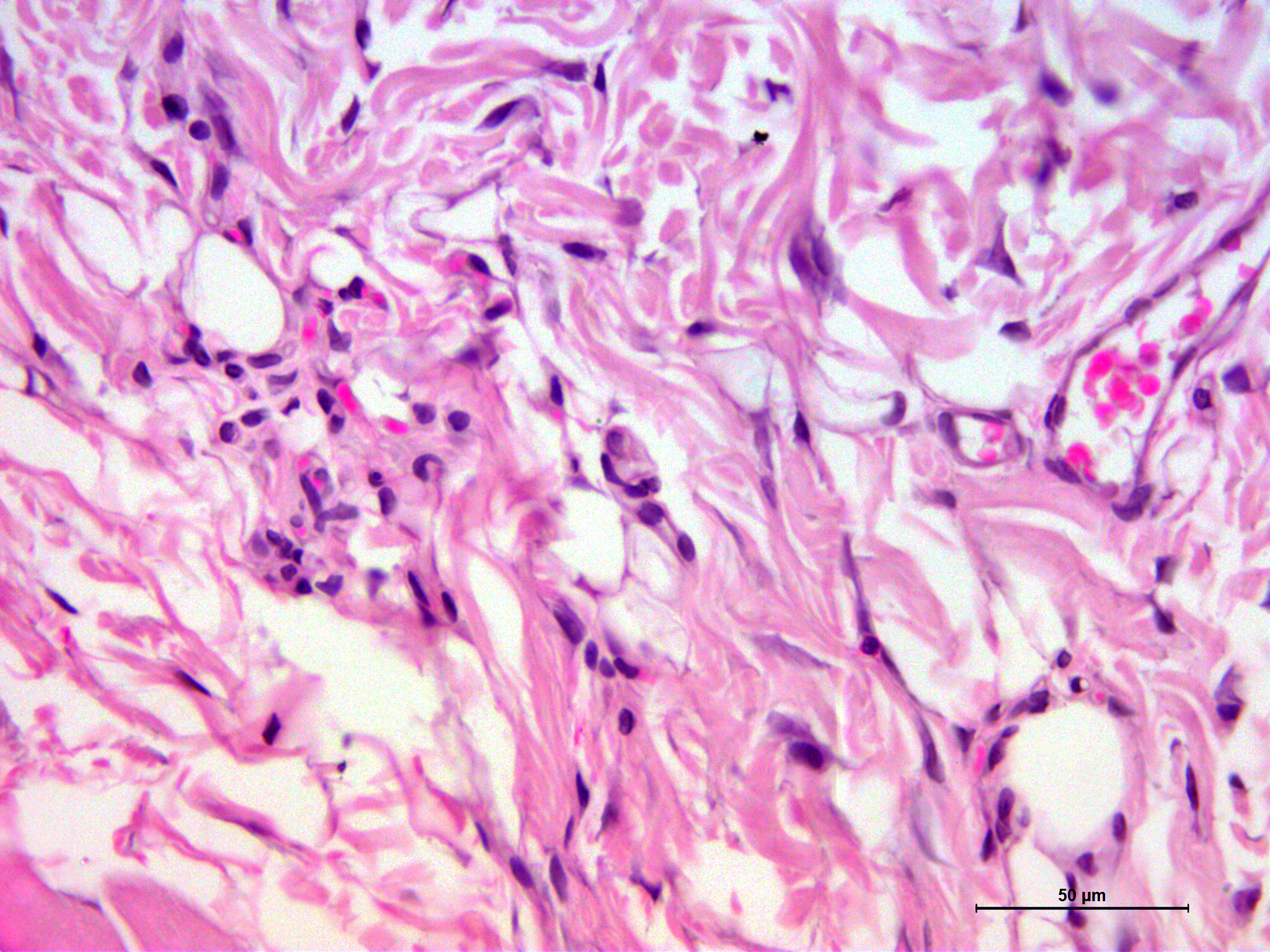

Supplement: Supplementary file 1 [file ijms-25-08631-s001.zip › Figure 1H (insert).tif]

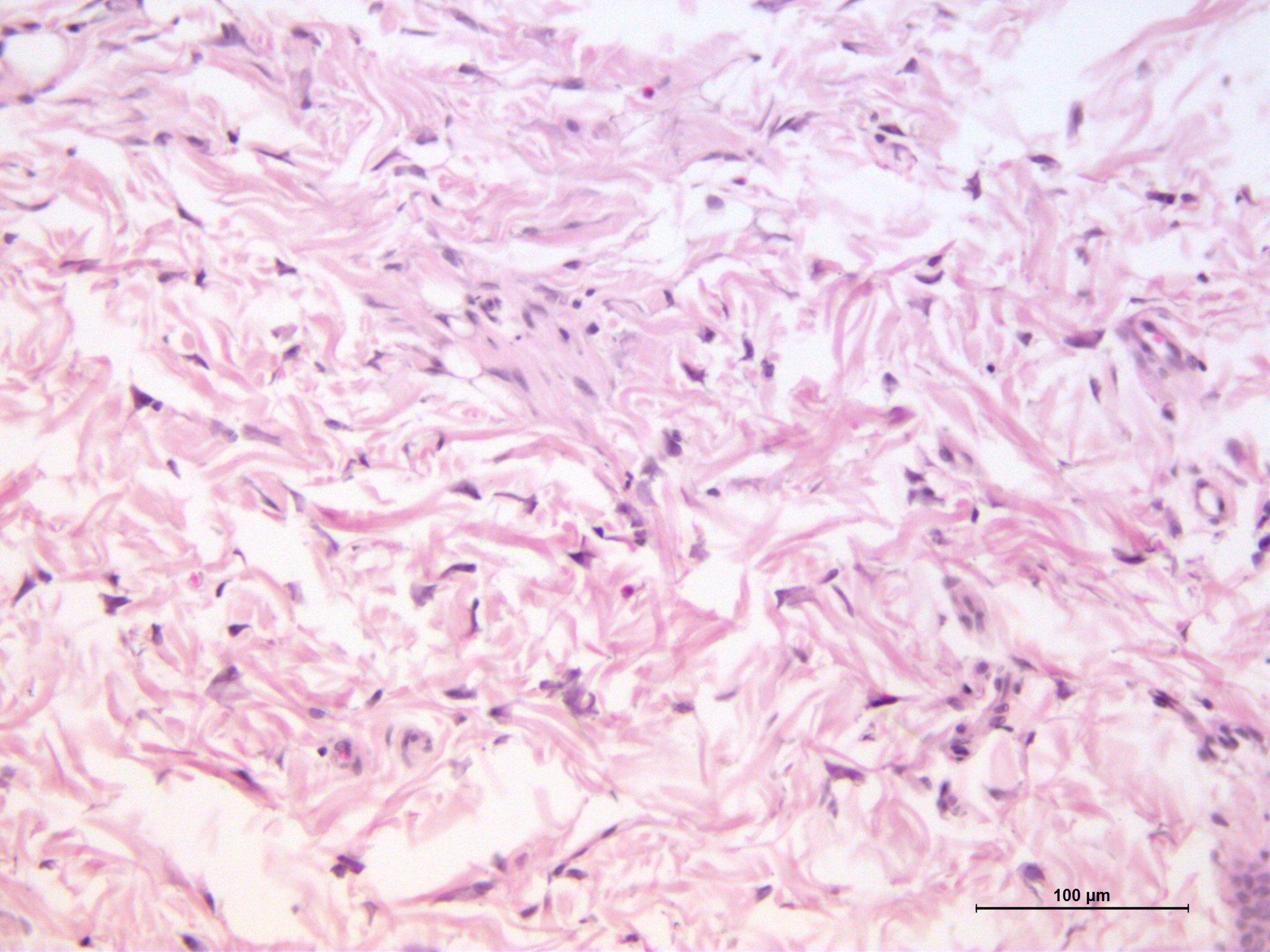

Supplement: Supplementary file 1 [file ijms-25-08631-s001.zip › Figure 1H.tif]

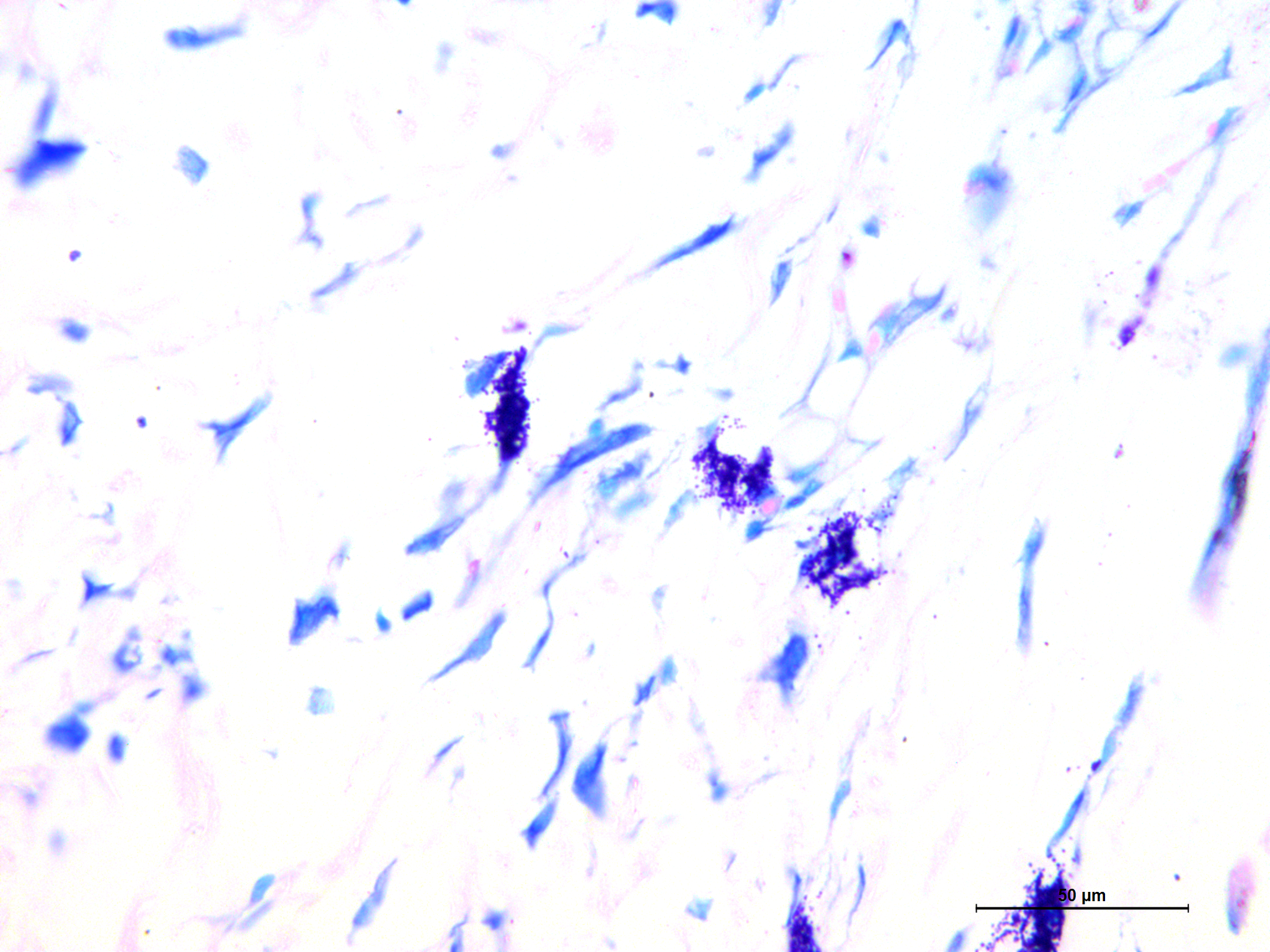

Supplement: Supplementary file 1 [file ijms-25-08631-s001.zip › Figure 1I (insert).tif]

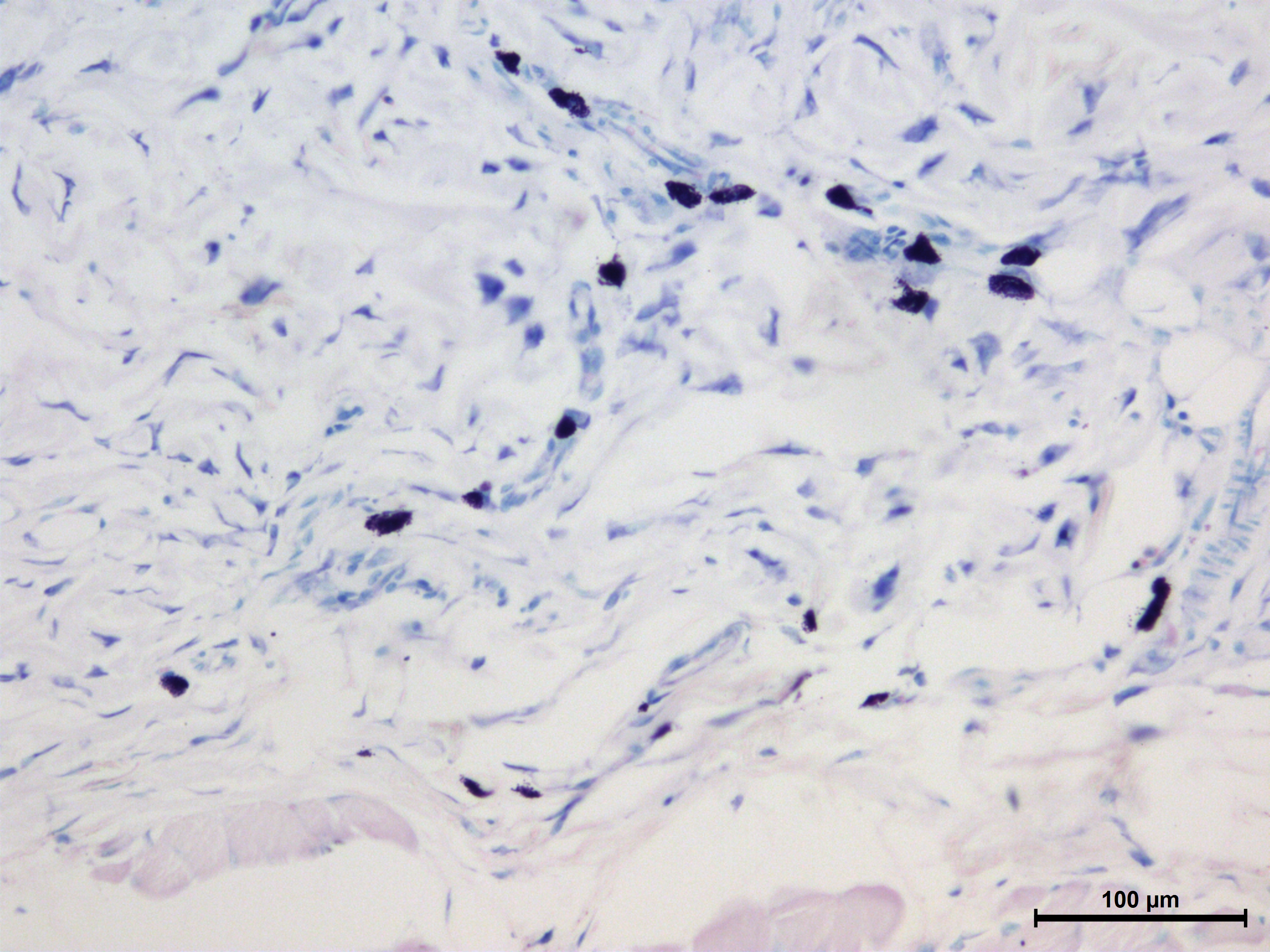

Supplement: Supplementary file 1 [file ijms-25-08631-s001.zip › Figure 1I.tif]
